# Effects of maternal type 1 diabetes and confounding factors on neonatal microbiomes

**DOI:** 10.1007/s00125-023-06047-7

**Published:** 2023-11-29

**Authors:** Marzena Gajecka, Pawel Gutaj, Katarzyna Jaskiewicz, Malgorzata Rydzanicz, Tomasz Szczapa, Dorota Kaminska, Grzegorz Kosewski, Juliusz Przyslawski, Rafal Ploski, Ewa Wender-Ozegowska

**Affiliations:** 1 Chair and Department of Genetics and Pharmaceutical Microbiology, Poznan University of Medical Sciences, Poznan, Poland; 2 Institute of Human Genetics, Polish Academy of Sciences, Poznan, Poland; 3 Department of Reproduction, Poznan University of Medical Sciences, Poznan, Poland; 4 Department of Medical Genetics, Medical University of Warsaw, Warsaw, Poland; 5 Department of Neonatology, Poznan University of Medical Sciences, Poznan, Poland; 6 Chair and Department of Bromatology, Poznan University of Medical Sciences, Poznan, Poland

**Keywords:** HbA_1c_, Maternal reproductive tract microbiome, Microbiome, Microbiota transfer, Neonatal microbiome, Type 1 diabetes

## Abstract

**Aims/hypothesis:**

Body niche-specific microbiota in maternal–neonatal dyads from gravidae with type 1 diabetes have not been quantitatively and functionally examined. Similarly, the impact of pregnancy-specific factors, such as the presence of comorbidities known to occur more frequently among gravidae with type 1 diabetes, including Caesarean delivery, as well as antibiotic prophylaxis, level of glycaemic control during each trimester of pregnancy and insulin administration, has not been adequately considered. The aims of this study were to characterise the maternal and neonatal microbiomes, assess aspects of microbiota transfer from the maternal microbiomes to the neonatal microbiome and explore the impact of type 1 diabetes and confounding factors on the microbiomes.

**Methods:**

In this observational case–control study, we characterised microbiome community composition and function using 16S rRNA amplicon sequencing in a total of 514 vaginal, rectal and ear-skin swabs and stool samples derived from 92 maternal–neonatal dyads (including 50 gravidae with type 1 diabetes) and in-depth clinical metadata from throughout pregnancy and delivery.

**Results:**

Type 1 diabetes-specific microbiota were identified among gravidae with type 1 diabetes and their neonates. Neonatal microbiome profiles of ear-skin swabs and stool samples were established, indicating the taxa more prevalent among neonates born to mothers with type 1 diabetes compared with neonates born to control mothers. Without taking into account the type 1 diabetes status of mothers, both delivery mode and intrapartum antibiotic prophylaxis were found to have an influence on neonatal microbiota composition (both *p*=0.001). In the logistic regression analysis involving all confounding variables, neonatal ear-skin microbiome variation was explained by maternal type 1 diabetes status (*p*=0.020) and small for gestational age birthweight (*p*=0.050). Moreover, in women with type 1 diabetes, a relationship was found between HbA_1c_ levels >55 mmol/mol (>7.2%) measured in the first trimester of pregnancy and neonatal ear-skin microbiota composition (*p*=0.008). In the PICRUSt (Phylogenetic Investigation of Communities by Reconstruction of Unobserved States) assessment, pathways concerning carbohydrate biosynthesis were predicted as key elements of the microbial functional profiles dysregulated in type 1 diabetes. Additionally, in SourceTracker analysis, we found that, on average, 81.0% of neonatal microbiota was attributed to maternal sources. An increase in the contribution of maternal rectum microbiota and decrease in the contribution of maternal cervix microbiota were found in ear-skin samples of vaginally delivered neonates of mothers with type 1 diabetes compared with neonates born to control mothers (83.2% vs 59.5% and 0.7% vs 5.2%, respectively).

**Conclusions/interpretation:**

These findings indicate that, in addition to maternal type 1 diabetes, glycaemic dysregulation before/in the first trimester of pregnancy, mode of delivery and intrapartum antibiotic prophylaxis may contribute to the inoculation and formation of the neonatal microbiomes.

**Data availability:**

The BioProject (PRJNA961636) and associated SRA metadata are available at http://www.ncbi.nlm.nih.gov/bioproject/961636. Processed data on probiotic supplementation and the PICRUSt analysis are available in the Mendeley Data Repository (https://doi.org/10.17632/g68rwnnrfk.1).

**Graphical Abstract:**

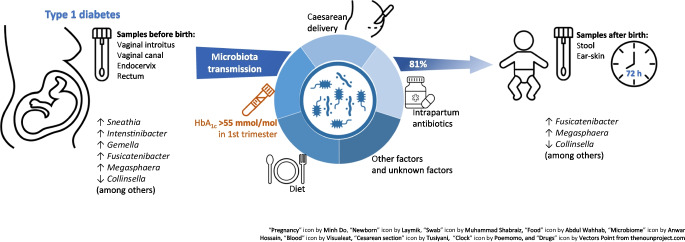

**Supplementary Information:**

The online version contains peer-reviewed but unedited supplementary material available at 10.1007/s00125-023-06047-7.


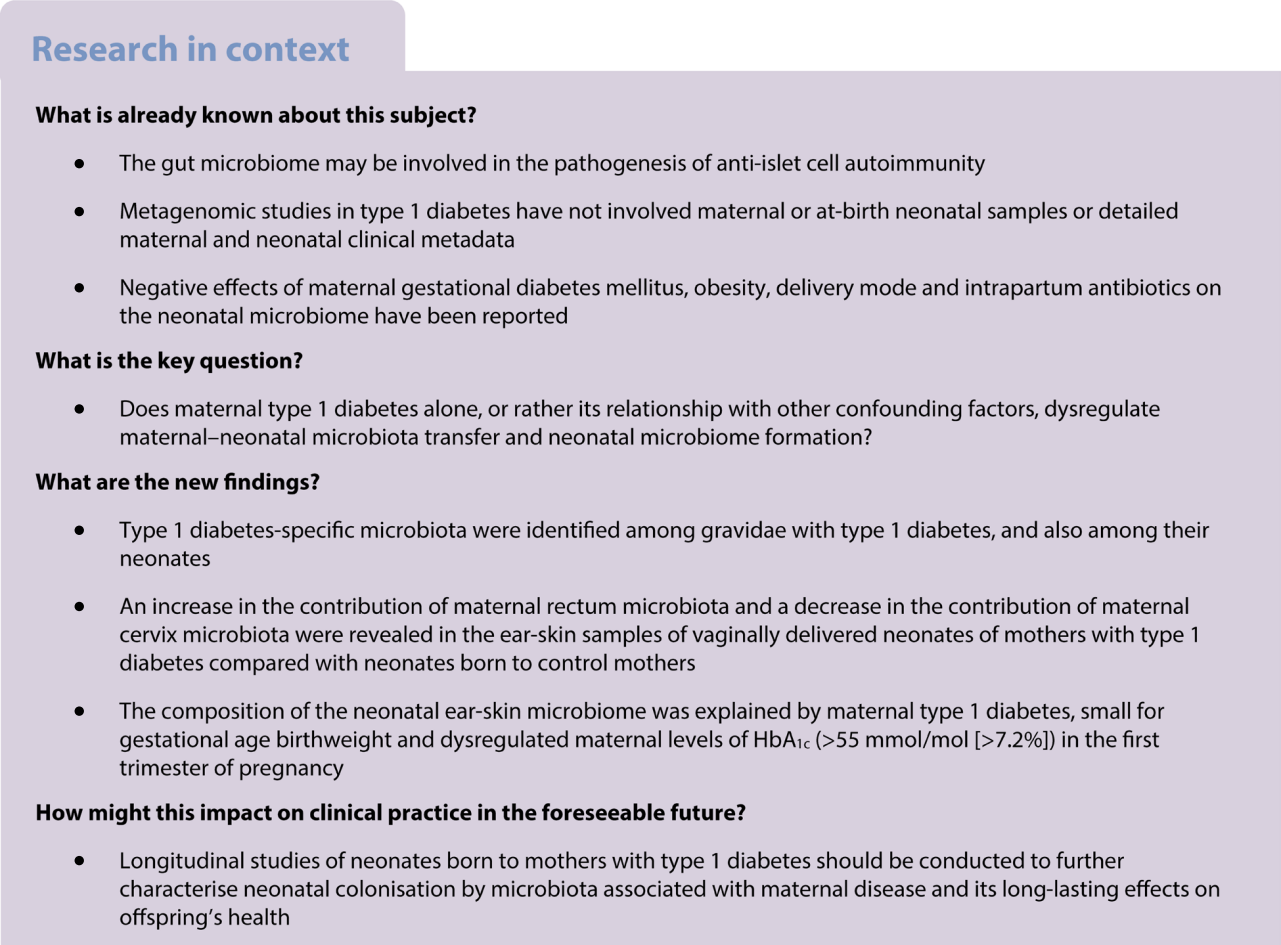


## Introduction

Type 1 diabetes mellitus is a chronic disease caused by insulin deficiency following autoimmune-mediated destruction of insulin-producing pancreatic beta cells. The disease complicates approximately 0.1–1% of all pregnancies [1–4], which is far less than the percentage complicated by either pre-existing type 2 diabetes or gestational diabetes mellitus (GDM) [3, 5]. Despite the relatively low prevalence of pregnancies in women with type 1 diabetes, type 1 diabetes accounts for a disproportionate burden of adverse pregnancy and neonatal outcomes, including a notably higher risk of miscarriage, preterm birth, preeclampsia, preterm delivery and macrosomia [1, 6]. Besides the known adverse pregnancy outcomes in type 1 diabetes, a shift towards a pro-inflammatory gut microbiome in pregnant women with type 1 diabetes has also been reported [7, 8]. In addition, outside pregnancy, type 1 diabetes is associated with dysregulation of gut and vaginal microbiomes [9, 10].

However, information on the effects of type 1 diabetes, type 2 diabetes and GDM on the neonatal microbiome is scarce. An early study showed lack of a negative impact of GDM on infants’ gut microbiome in the first years of life [11]. This was followed by studies reporting an association between vaginal dysbiosis in GDM and adverse perinatal outcomes [12], an imbalance of gut microbiota in neonates born to non-diabetic [13] and GDM mothers [14] with obesity, and differences in meconium microbiome composition in full-term newborns delivered by Caesarean section to GDM mothers [15]. The Environmental Determinants of Diabetes in the Young (TEDDY) study [16, 17] pointed to a minor role, if any, for infant or early childhood microbiomes in the aetiology of type 1 diabetes. These findings were in contrast to earlier and smaller cohort studies that reported that a permeable intestinal mucosal barrier and an altered mucosal immune response collectively contribute to the development of type 1 diabetes [18] and that the microbiome itself may be involved in the pathogenesis of anti-islet cell autoimmunity [19]. In multicentre studies exploring determinants of type 1 diabetes/islet autoimmunity (TEDDY, All Babies In Southeast Sweden [ABIS] and Australia-wide Environmental Determinants of Islet Autoimmunity [ENDIA] studies), the effect of maternal type 1 diabetes on neonatal microbiomes has not been studied and analysis in mother–neonate dyads has not been performed [8, 16, 17, 20].

Here we investigate the effects of maternal disease alone and confounding factors on both maternal and offspring microbiomes, assessing microbiota profiles of the vaginal introitus, vaginal canal, endocervix and rectum and neonatal stool samples and ear-skin swabs. To minimise the influence of environmental and methodological factors [21, 22], the study included gravidae residing in the same region of Poland, of the same ethnic origin and giving birth in the same obstetrical hospital; all experimental samples were also subjected to the same sequencing experiment. We hypothesised that dysregulation of HbA_1c_ levels in the first trimester may have long-term consequences and may influence the composition of the neonatal microbiome. Gravidae with type 1 diabetes with satisfactory glycaemic control in the second and third trimesters of pregnancy were investigated; therefore, the number of factors affecting the interpretation of the results was limited. We also hypothesised that type 1 diabetes itself and its relationship with other confounding factors dysregulate inoculation (maternal–neonatal microbiota transfer) and the formation of neonatal microbiomes.

In this study we used a comprehensive approach to characterise the composition of maternal and neonatal microbiomes, assess aspects of maternal–neonatal microbiota transfer and explore the impact of type 1 diabetes and variables such as Caesarean section and antibiotic prophylaxis on neonatal microbiomes. We also investigated key elements of the microbial functional profiles that are dysregulated in type 1 diabetes.

## Methods

### Participant ascertainment

Gravidae with type 1 diabetes and control gravidae without type 1 diabetes and their neonates were ascertained in the Gynecologic and Obstetrical University Hospital at the Poznan University of Medical Sciences, which is publicly available to patients (see electronic supplementary material [ESM] Methods for details, ESM Fig. 1 for details of the recruitment procedure and Table 1 and ESM Table 1 for participant characteristics). Socioeconomic factors were not investigated.

**Table 1:** Selected clinical characteristics of women with type 1 diabetes (T1D) and control women

Clinical characteristic	T1D group (*n*=50)	Control group (*n*=42)	*p* value
Age (years), mean ± SD	30.5±4.1	32.4±3.7	0.011
Age at delivery (full years), mean ± SD	30±4	32±4	0.008
Ethnicity: White^a^	50 (100.0)	42 (100.0)	
Geographical location: Poland	50 (100.0)	42 (100.0)	
Delivery mode			
Caesarean	33 (66.0)	20 (47.6)	0.118
Unscheduled	2 (4.0)	2 (4.8)	
Scheduled	31 (62.0)	18 (42.9)	
Vaginal delivery	12 (24.0)	17 (40.5)	
Vacuum-assisted vaginal delivery	5 (10.0)	5 (11.9)	
Gestation (weeks), mean ± SD	38.1±0.7	39.2±1.0	<0.001
Pre-pregnancy BMI (kg/m^2^), mean ± SD^b^	23.19±2.95	22.46±2.56	0.423
Pre-pregnancy BMI^b^			
Underweight	1 (2.0)	1 (2.4)	0.561
Normal	35 (70.0)	33 (78.6)	
Overweight	13 (26.0)	7 (16.7)	
Obese	1 (2.0)	0 (0.0)	
NA	0 (0.0)	1 (2.4)	
Before-delivery BMI (kg/m^2^), mean ± SD^b^	28.12±3.75	27.46±2.97	0.649
Weight gain during pregnancy (kg), mean ± SD^b^	13.61±5.34	14.00±3.79	0.727
Weight gain^c^			
Non-excessive	32 (64.0)	26 (61.9)	0.954
Excessive	18 (36.0)	15 (35.7)	
NA	0 (0)	1 (2.4)	
HbA_1c_ levels met targets during pregnancy^d^			
Yes	50 (100.0)	0 (0.0)	–
No	0 (0.0)	42 (100.0)	–
Age at T1D diagnosis (years), mean ± SD	17.91±8.21	NA	–
Duration of T1D (years), mean ± SD	12.56±7.02	NA	–
Diabetes medication (insulin therapy)			
Yes	50 (100.0)	0 (0.0)	–
No	0 (0.0)	42 (100.0)	–
Time on insulin therapy (years), mean ± SD	12.52±17.09	NA	–
Age at beginning of insulin therapy (years), mean ± SD	17.97±8.30	NA	–
Classification of pregestational diabetes (modified P. White classification)^c^			
Class A	0 (0.0)	NA	–
Class B	18 (36.0)	NA	–
Class C	15 (30.0)	NA	–
Class D	11 (22.0)	NA	–
Class R	5 (10.0)	NA	–
Class F	0 (0.0)	NA	–
Class RF	1 (2.0)	NA	–
Class H	0 (0.0)	NA	–
Class T	0 (0.0)	NA	–
Retinopathy			
Yes	12 (24.0)	0 (0.0)	0.002
No	38 (76.0)	42 (100.0)	
Antibiotic prophylaxis			
Yes	34 (68.0)	20 (47.6)	0.048
No	16 (32.0)	22 (52.4)	
Reason for antibiotic prophylaxis^e^			
Caesarean section	28 (80.0)	12 (54.5)	0.083
*Streptococcus agalactiae*	5 (14.3)	5 (22.7)	
Chorioamnionitis (intra-amniotic infection)	0 (0.0)	0 (0.0)	
Maternal bacterial endocarditis	0 (0.0)	0 (0.0)	
Premature rupture of membranes	2 (5.7)	5 (22.7)	
Antibiotic used^f^			
Cefuroxime	18 (51.4)	7 (30.4)	0.385
Ampicillin	6 (17.1)	4 (17.4)	
Cefazolin	11 (31.4)	10 (43.5)	
Clindamycin	0 (0.0)	1 (4.3)	
Cefalexin	0 (0.0)	1 (4.3)	
Any chronic disease/disorder (excluding T1D)^g^			
Yes	6 (12.0)	1 (2.4)	<0.001
No	44 (88.0)	41 (97.6)	
Hypertension			
Yes	3 (6.0)	0 (0.0)	0.112
No	47 (94.0)	42 (100.0)	
Gestational hypertension			
Yes	2 (4.0)	1 (2.4)	0.663
No	48 (96.0)	41 (97.6)	
Maternal preeclampsia			
Yes	2 (4.0)	0 (0.0)	0.195
No	48 (96.0)	42 (100.0)	
Processed samples^h^	50 (100.0)	41 (97.6)	

Data are *n* (%) unless reported otherwise

Statistical significance is based on the two-tailed *χ*^2^ test and two-tailed Mann–Whitney *U* test for qualitative and quantitative data, respectively

An extended version of Table 1 is provided as ESM Table 1

^a^Self-reported by participants

^b^Weight before pregnancy was self-reported and BMI was calculated by a researcher; weight and BMI just before delivery were measured and calculated in the hospital

^c^According to Wender-Ozegowska et al [34]

^d^HbA_1c_ measurement in each of the three trimesters of pregnancy

^e^In one T1D participant and two control participants there was more than one reason for antibiotics prophylaxis

^f^In one T1D participant and three control participants more than one antibiotic was administered

^g^Hypertension, asthma or epilepsy

^h^Reasons for not processing samples were incomplete collection of biological sample sets from participants or poor quality of the samples

NA, not applicable; T1D, type 1 diabetes

#### Inclusion criteria

The inclusion criteria for both study groups were age 18–45 years and single, term pregnancy (37+0 to 41+0). The exclusion criteria were multiple pregnancies, genitourinary infection diagnosed in the last 4 weeks, administration of oral or vaginal antibiotics/antifungal medicines/probiotic supplementation in pregnancy in the previous 4 weeks, and vaginal irrigation/sexual intercourse in the last 72 h.

According to national guidelines [23], all women from the control group were screened for hyperglycaemia in pregnancy and were free of diabetes mellitus in pregnancy or GDM (see ESM Methods). Details of the assessment of HbA_1c_ levels are provided in ESM Methods.

#### Nutritional assessment

The ascertained women underwent detailed nutritional assessment using 24 h dietary recall for 7 days. In addition, information about the participants was collected based on a survey developed by the authors (see ESM Methods) and the validated Food Frequency Questionnaire FFQ-D10 [24] (see ESM Methods).

Clinical information concerning the mothers and their neonates was collected during the clinic stay and included the mode of birth, the Apgar score, pregnancy complications, information about maternal type 1 diabetes, gestational age, any medication taken during pregnancy, feeding method and comorbidities.

#### Ethics permission

The study was approved by the Institutional Review Board at the Poznan University of Medical Sciences (no. 132/13, 223/13, 241/13, 194/14). The mothers provided written informed consent.

### Sample collection, processing and storage

Three swabs from the genital tract and one from the rectum of gravidae (eSwab Liquid Amies Collection and Transport System, Copan Diagnostics, USA) were collected by the obstetrician immediately after administration to the hospital to avoid sample contamination by the environment. These two sampling sites were evaluated because of the anatomical proximity of the rectum and vaginal introitus, and to assess both microbiota allocation in mothers and mother–neonate microbiota transfer during vaginal delivery. We collected the microbiome from three sites in the reproductive tract (vaginal introitus, middle of the vaginal canal, and cervix) to characterise the diversity of microbiota as well as further investigate the mother–neonate microbiota transfer. Neonatal samples were collected up to 72h after the birth. A swab from neonatal ear-skin was collected by a neonatologist. Neonatal stool samples, other than the first stool, were collected from a diaper on the second or third day postpartum. A total of 530 samples were collected. The material was stored at −80°C before further processing.

### 16S rRNA gene sequencing and data analysis

Of the 530 DNA samples, 514 met quantitative and qualitative criteria for sequencing. The 16S rRNA gene sequencing method used and bioinformatics analyses carried out are described in ESM Methods. After quality control of the reads generated, 511 samples were retained for analysis.

In each sampling site we computed the mean abundance of taxa compared with all sites, multiplied by the frequency of appearance of these taxa (abundance >0.0%) across all samples within the site. Bubble plots of relative taxonomic abundances were generated using the R package ggplot2 (v3.3.5) [25]. Microbiota diversity within samples (alpha diversity, represented by the Shannon diversity index) and between samples (beta diversity, represented by the weighted UniFrac dissimilarity index) was evaluated based on the amplicon sequence variants (ASV). The Wilcoxon test on Shannon diversity indexes was applied. For beta diversity measurements, principal coordinates analysis (PCoA) plots were generated to visualise the compositional diversity, whereas statistical significance (*p* values) was calculated using adonis2 permutational analysis of variance (PERMANOVA; permutations = 9999). For details of 16S rRNA sequencing data analysis, neonatal microbiota profiles and PICRUSt (Phylogenetic Investigation of Communities by Reconstruction of Unobserved States) analysis (prediction of metagenome function), see ESM Methods.

#### Effect of glycaemic control on microbiota composition

To assess the effect of long-term glycaemic control on microbiota composition we compared the relative abundance of all genera based on weighted UniFrac dissimilarity indices in two subgroups of women with type 1 diabetes (HbA_1c_ ≤55 mmol/mol [≤7.2%] vs >55 mmol/mol [>7.2%]). HbA_1c_ levels in the first, second and third trimesters were considered in the analyses. For details see ESM Methods.

#### SourceTracker2 Analysis

SourceTracker2 [26] analysis was performed to predict the origin of neonatal microbiota using the maternal microbiota as potential sources; this was evaluated by disease state and delivery mode. The ASV and taxonomy tables were imputed using default parameters, with the maternal samples defined as the ‘source’ and the neonatal samples defined as the ‘sink’. ANOVA with a Tukey’s honestly significant difference (HSD) post hoc test was used for statistical analysis of the microbial contributions obtained.

### Statistical analyses

Except where noted, all statistical analyses were performed using R (v3.4.4) [27]. To adjust for multiple comparisons, permutational testing, Tukey’s HSD correction, Benjamini and Hochberg correction or the false discovery rate (FDR) based method was performed. The R packages ggpubr (v0.4.0) [28], pheatmap (v1.0.12) [29], vegan (v2.5-7) [30], phyloseq (v1.22.3) [31] and ggplot2 (v3.3.5) [25] were used to perform and visualise the analyses of the clinical/metagenomic data.

## Results

### Clinical characteristics of maternal–neonatal dyads

Fifty gravidae with type 1 diabetes and their neonates, and 42 control gravidae and their neonates were ascertained (ESM Fig. 1).

The clinical characteristics of gravidae with type 1 diabetes and control gravidae are presented in Table 1, ESM Table 1 and ESM Fig. 2. Compared with control women, women with type 1 diabetes were more likely to deliver prior to 39 weeks (*p*<0.001) (ESM Fig. 2a), and were younger at delivery (*p*=0.008).. There was no statistically significant difference in terms of delivery mode comparing the ratio of Caesarean to vaginal deliveries in pregnancies affected by type 1 diabetes and in control gravidae. Indications for Caesarean section in gravidae are described in ESM Results.

BMI before pregnancy, weight gain during pregnancy and BMI before delivery were comparable between the two groups of women (Table 1). Compared with control women, women with type 1 diabetes had a higher energy intake from proteins and a lower energy intake from carbohydrates (*p*=0.030 and *p*=0.017, respectively; ESM Table 1). Detailed dietary information obtained for the studied women is described elsewhere [32]. In our cohort, adequate glycaemic control (as measured by HbA_1c_ ≤43 mmol/mol [≤6.1%], ESM Fig. 2b) was observed in the second and third trimesters in women with type 1 diabetes.

The clinical characteristics of the neonates are presented in Table 2. No preterm neonates were involved in the study. In total, 64% vs 93%, 28% vs 7% and 8% vs 0% of the neonates born to women with type 1 diabetes vs control women were classified as appropriate, large and small for gestational age, respectively (AGA, LGA and SGA, respectively; *p*=0.01; Table 2, ESM Fig. 3a) in accordance with regional growth charts [33].

**Table 2:** Clinical characteristics of neonates delivered by women with type 1 diabetes (T1D) and control women

Clinical characteristic	T1D group (*n*=50)	Control group (*n*=42)	*p* value
Sex			
Male	27 (54.0)	24 (57.1)	0.763
Female	23 (46.0)	18 (42.9)	
Birthweight			
SGA	4 (8.0)	0 (0.0)	0.010
AGA	32 (64.0)	39 (92.9)	
LGA	14 (28.0)	3 (7.1)	
Birthweight (kg), mean ± SD	3552.80±524.26	3405.45±395.51	0.095
Feeding method			
Breast milk	7 (14.0)	40 (95.2)	<0.001
Formula	0 (0.0)	2 (4.8)	
Mixed	41 (82.0)	0 (0.0)	
ND	2 (4.0)	0 (0.0)	
Day that formula was introduced into the diet, mean ± SD	1±0	1±0	
Monitoring of postnatal glucose homeostasis			
Yes	49 (98.0)	0 (0.0)	
No	0 (0.0)	42 (100.0)	
ND	1 (2.0)	0 (0.0)	
Postnatal glucose level (mmol/l), mean ± SD^a^	2.80±1.06	NA	
Processed samples^b^	48 (96.0)	41 (97.6)	

Data are *n* (%) unless otherwise reported

Statistical significance is based on the two-tailed *χ*^2^ test and two-tailed Mann–Whitney *U* test for qualitative and quantitative data, respectively

^a^Measured in the first hour of life

^b^The reason for not processing samples was incomplete collection of biological sample sets from participants

NA, not available; ND, no data; T1D, type 1 diabetes

No hypoglycaemia was observed in the neonates born to women with type 1 diabetes (ESM Fig. 3b,c) and no correlation was found between HbA_1c_ levels in women with type 1 diabetes measured before delivery and glucose levels in their offspring (ESM Fig. 4).

### Maternal microbiome community composition and bacteria associated with type 1 diabetes

**Fig. 1:**
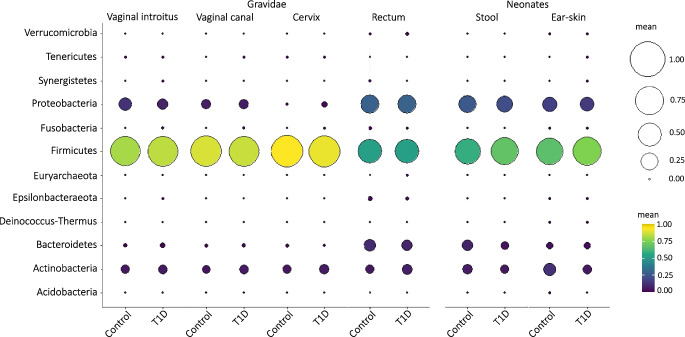
Characterisation of the maternal and neonatal microbiome using targeted 16S rRNA gene amplicon sequencing. The mean relative abundance of the 12 most prevalent phyla (Verrucomicrobia, Tenericutes, Synergistetes, Proteobacteria, Fusobacteria, Firmicutes, Euryarchaeota, Epsilonbacteraeota, Deinococcus-Thermus, Bacteroidetes, Actinobacteria and Acidobacteria) in the maternal vaginal introitus (type 1 diabetes, *n*=50 vs control, *n*=41), vaginal canal (type 1 diabetes, *n*=50 vs control, *n*=41), cervix (type 1 diabetes, *n*=50 vs control,* n*=41) and rectum (type 1 diabetes, *n*=44 vs control, *n*=40) as well as neonatal ear-skin swabs (type 1 diabetes, *n*=42 vs control, *n*=39) and stool samples (type 1 diabetes, *n*=42 vs control, *n*=31) are shown. The Firmicutes, Proteobacteria, Actinobacteria and Bacteroidetes dominated among the 12 phyla examined. Differences in the relative abundance of phyla in the three vaginal sampling sites were found (statistical significance based on Wilcoxon rank sum test). A low variability of bacteria near the cervix and a pattern of increasing abundance for the phylum Firmicutes, increasing from the introitus through the centre of the vagina to the cervix, were found in both women with type 1 diabetes and control women (introitus vs cervix: *p*=0.087). Proteobacteria dominated the introitus of the vagina of both women with type 1 diabetes and control women compared with other sampling sites (*p*=0.001) and were almost absent in the cervix, while the relative abundance of Actinobacteria and Bacteroidetes were comparable (*p*>0.05) in the vaginal sampling sites assessed. No substantial differences in relative abundance of phyla in the samples derived from neonates were found. T1D, type 1 diabetes

The three maternal reproductive tract sampling sites (vaginal introitus, vaginal canal and cervix) showed a similar abundance of bacteria but a notably lower bacterial abundance than the rectal sampling site, as demonstrated by the alpha diversity indices (for details see the Shannon index values in ESM Fig. 5a). Comparing women with type 1 diabetes and control women, no substantial differences in bacterial abundance (alpha diversity) were found between any of the maternal sampling sites studied (ESM Fig. 5a). However, substantial differences (*p*=0.041) in microbial composition (beta diversity) of the rectum swabs between the women with type 1 diabetes and the control women were found (see beta diversity PCoA plots in ESM Fig. 5b). The delivery week (37–41) affected the microbial composition (*p*=0.002) of the samples assessed (ESM Fig. 6). The 12 most prevalent phyla in the three vaginal sampling sites were compared between women with type 1 diabetes and control women (Fig. 1) as described in detail in ESM Results.

**Fig. 2:**
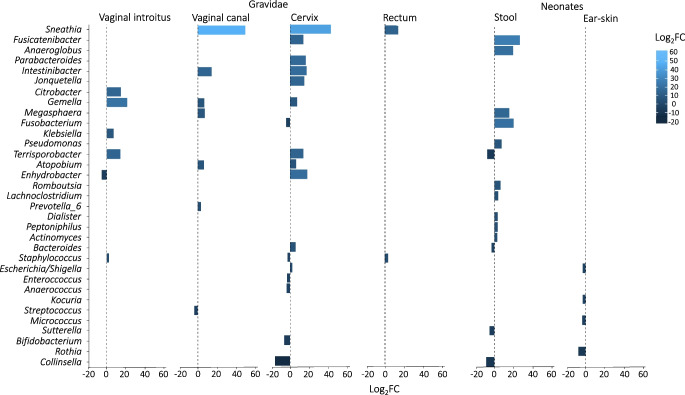
Bacteria associated with type 1 diabetes in mothers (differentially expressed bacterial genera) revealed as over-represented or under-represented in the maternal vaginal introitus (type 1 diabetes, *n*=50 vs control, *n*=41), vaginal canal (type 1 diabetes, *n*=50 vs control, *n*=41), cervix (type 1 diabetes, *n*=50 vs control, *n*=41) and rectum (type 1 diabetes, *n*=44 vs control, *n*=40) as well as neonatal stool samples (type 1 diabetes,* n*=42 vs control, *n*=31) and ear-skin swabs (type 1 diabetes, *n*=42 vs control,* n*=39) samples. The plot shows log_2_ fold change (log_2_FC) values, which demonstrate the direction of change in the relative abundance of particular genera associated with type 1 diabetes. All genera presented showed statistically significant variation in bacterial relative abundance (statistical significance based on the Wald test with Benjamini and Hochberg correction for multiple testing). Among the 17 genera in the cervix of women with type 1 diabetes, 11 were over-represented and six were under-represented. In the vaginal introitus and vaginal canal, the numbers of differentially expressed bacteria associated with type 1 diabetes were similar. Two genera, *Staphylococcus* and *Sneathia*, were over-represented in the samples taken from the rectum of women with type 1 diabetes. No over-represented bacteria associated with type 1 diabetes were identified in ear-skin swabs. *Rothia*, *Micrococcus*, *Escherichia/Shigella* and *Kocuria* were under-represented in these samples. *Fusicatenibacter*, *Fusobacterium*, *Anaeroglobus*, *Megasphaera*, *Pseudomonas*, *Romboutsia*, *Lachnoclostridium, Dialister*, *Peptoniphilus* and *Actinomyces* were over-represented in the stool samples derived from neonates of women with type 1 diabetes, while *Collinsella*, *Terrisporobacter*, *Sutterella* and *Bacteroides* were under-represented in these samples. See ESM Table 2 and ESM Fig. 7 for a full list of observed differences

In a more detailed analysis, bacteria specific to type 1 diabetes (differentially expressed genera) were identified in the maternal sampling sites, with the majority of these bacteria (17 genera) found in the cervix (Fig. 2, ESM Fig. 7, ESM Table 2). *Sneathia*, *Gemella*, *Staphylococcus*, *Intestinibacter*, *Atopobium*, *Terrisporobacter* and *Enhydrobacter* were associated with type 1 diabetes in more than one maternal sampling site (ESM Table 2).

### Neonatal microbiome community composition and bacteria associated with type 1 diabetes

A lower bacterial abundance (alpha diversity) was found in the neonatal stool samples than the maternal rectal samples, whereas the neonatal ear-skin samples showed a higher bacterial abundance than the neonatal stool samples (for details see the Shannon index values in ESM Fig. 5a). There were no statistically significant differences in alpha diversity estimates for ear-skin swabs or stool samples between neonates born to women with type 1 diabetes and those born to control women (ESM Fig. 5a).

In contrast to the ear-skin swabs, there were differences in the microbial composition (beta diversity) of stool samples between the neonates of women with type 1 diabetes and the neonates of control women, although these were not statistically significant (*p*=0.054; see beta diversity PCoA plots in ESM Fig. 5b). Additionally, we evaluated the possible influence of sampling time on the composition of the neonatal stool microbiome but found no differences (results not shown).

No substantial differences in the relative abundance of phyla in the samples derived from neonates were found. Firmicutes, Proteobacteria, Actinobacteria and Bacteroidetes dominated among the 12 most prevalent phyla. The relative abundance of Bacteroidetes in stool samples from neonates delivered by control women was similar to that found in rectum swabs from their mothers (Fig. 1).

Next, bacteria associated with type 1 diabetes in the mothers were assessed in the neonatal samples (Fig. 2, ESM Fig. 7, ESM Table 2). Seven of the genera associated with type 1 diabetes found in maternal samples (*Bacteroides*, *Collinella*, *Escherichia/Shigella*, *Fusicatenibacter*, *Fusobacterium*, *Megasphaera*, *Terrisporobacter*) were also revealed as specific in the ear-skin swabs or stool samples from their offspring.

A comparison between the taxa that were more prevalent among neonates of mothers with type 1 diabetes and the taxa that were more prevalent among neonates of control mothers is shown in ESM Fig. 8 and ESM Table 3.

### Differences in microbiota composition caused by confounding variables

Both the mother's type 1 diabetes status and the mode of delivery influenced the composition of the neonatal microflora in the univariate analysis (*p*=0.025 and *p*=0.001, respectively; ESM Table 4). However, in the multivariate analysis, these two variables did not reach statistical significance (*p*>0.05; ESM Table 4).

In addition, intrapartum antibiotic prophylaxis was found to influence the microbiota composition in neonates (*p*=0.001; ESM Table 4). Further, the composition of microbiota in neonatal samples differed between the two groups taking into account both antibiotic prophylaxis and the maternal type 1 diabetes disease status (*p*=0.006; ESM Table 4). Further details of the impact of antibiotic prophylaxis on bacterial diversity in mothers are provided in ESM Results.

**Fig. 3:**
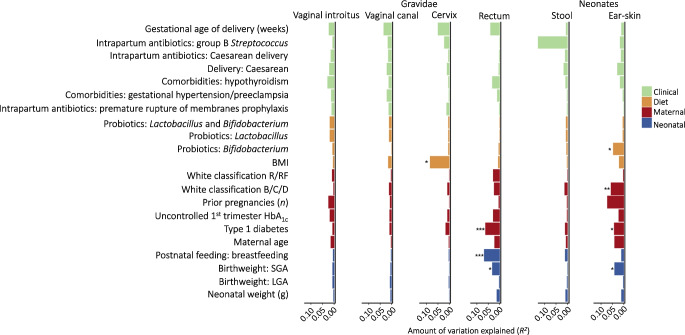
Microbiome variation explained by all confounding variables evaluated in the maternal vaginal introitus (type 1 diabetes, *n*=50 vs control, *n*=41), vaginal canal (type 1 diabetes, *n*=50 vs control, *n*=41), cervix (type 1 diabetes, *n*=50 vs control, *n*=41) and rectum (type 1 diabetes, *n*=44 vs control, *n*=40) as well as neonatal stool samples (type 1 diabetes, *n*=42 vs control, *n*=31) and ear-skin swabs (type 1 diabetes, *n*=42 vs control, *n*=39). The goodness-of-fit statistic *R*^2^, used to determine the percentage of microbiome variation explained by a variable, was calculated using multiple regression of environmental vectors and factors as dependent variables against ordination axes of variance stabilising transformation (VST)-transformed Euclidean sample distances as explanatory variables. To adjust for multiple comparisons, the significance of the correlation coefficients was tested using Monte Carlo permutational testing (*n*=999). See ESM Table 5 for a full list of the observed differences. Across neonatal ear-skin samples, the microbiome variation was explained by the following covariates: maternal use of probiotics containing *Bifidobacterium*, maternal type 1 diabetes status, severity/duration of maternal disease (White classification B/C/D for type 1 diabetes) and SGA birthweight. The covariates with statistical significance for maternal rectum samples were type 1 diabetes status, breastfeeding and SGA birthweight. For maternal cervix samples, pre-pregnancy BMI was the main explanatory covariate for microbiome variation. *p≤0.05, **p≤0.01, ***p≤0.001. B, pregestational diabetes onset after 20 years of age or duration <10 years; C, pregestational diabetes onset between 10 and 19 years of age or duration of 10–19 years; D, pregestational diabetes onset before 10 years of age or duration >20 years or retinopathy or hypertension; R, pregestational diabetes with proliferative retinopathy or haemorrhage to the vitreous; RF, pregestational diabetes with proliferative retinopathy or haemorrhage to the vitreous, nephropathy and proteinuria before pregnancy >0.5 g/24 h

Finally, in logistic regression analysis involving all confounding variables, maternal type 1 diabetes status was found to explain the variation in neonatal ear-skin and maternal rectal microbiomes (*p*=0.020 and *p*<0.001, respectively). Moreover, the following variables showed statistical significance in this multivariate model: severity/duration of maternal disease (White classification B/C/D [34]), SGA birthweight, maternal use of probiotics containing *Bifidobacterium,* breastfeeding and pre-pregnancy BMI, as presented in Fig. 3 and ESM Table 5.

The results on the impact of selected dietary aspects on maternal microbiomes, microbial functional profiles dysregulated in type 1 diabetes (PICRUSt analysis) and breastfeeding are provided in ESM Results, ESM Table 6 and ESM Figs 9 and 10.

### Impact of glycaemic control on the maternal and neonatal microbiome communities

In accordance with HbA_1c_ dysregulation in the first trimester of pregnancy in women with type 1 diabetes (ESM Fig. 2b), we assessed the microbiota community composition in two subgroups of women with type 1 diabetes (HbA_1c_ ≤55 mmol/mol [≤7.2%] vs HbA_1c_ >55 mmol/mol [>7.2%]); this threshold value was found to be discriminatory in our metadata (ESM Fig. 11, ESM Table 7). Overall, HbA_1c_ levels, which were satisfactory in the second and third trimesters in the majority of gravidae (ESM Fig. 2b), did not influence the maternal microbiota communities sampled just before delivery. However, a relationship was found between HbA_1c_ levels >55 mmol/mol (>7.2%) measured in the first trimester of pregnancy in gravidae with type 1 diabetes and neonatal ear-skin microbiota (*p*=0.008; ESM Fig. 11, ESM Table 7).

### Computed microbiota transfer from maternal to neonatal microbiomes

**Fig. 4:**
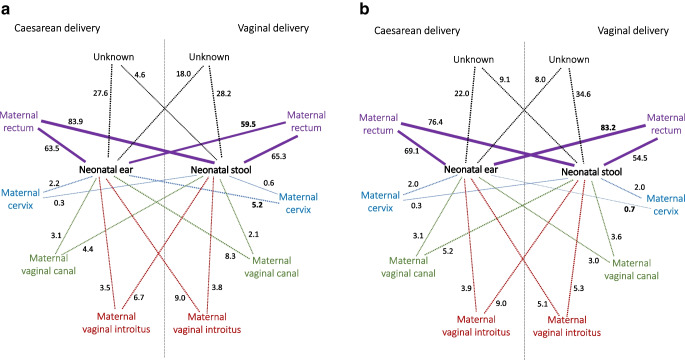
Contribution of maternal microbiomes originating from the vaginal introitus, vaginal canal, cervix and rectum (considered ‘sources’ in the microbiota transfer, presented as outside nodes) to microbial communities in neonates (stool samples and ear-skin swabs, considered ‘sinks’ in the microbiota transfer, presented as inside nodes). SourceTracker2-derived contributions evaluated by maternal type 1 diabetes status and delivery mode: (**a**) control (*n*=31) and (**b**) type 1 diabetes (*n*=42). The numbers on the lines represent mean contributions (%). Mean values are in the range 0.3–83.9% (100% represents full contribution from a source to a sink, whereas 0% represents no contribution). A statistically significant (ANOVA with a Tukey’s HSD post hoc test, *p*<0.05) increase in maternal contributions to the neonatal ear-skin microbiome from the rectum and a decrease in contributions from the cervix were found when comparing vaginally delivered neonates of mothers with type 1 diabetes with vaginally delivered neonates born to control mothers. See ESM Table 8 for a full list of observed differences

SourceTracker2 software was used to estimate the contribution of maternal microbiomes originating from the vaginal introitus, vaginal canal, cervix and rectum (considered the microbiota ‘sources’ in the transfer) to microbial communities in neonates (stool samples and ear-skin swabs, considered the ‘sinks’ in the transfer), evaluated by disease status and delivery mode. The results are presented in Fig. 4 and ESM Table 8. On average, 81.0% of neonatal microbiota were attributed to maternal sources, of which 69.0% were assigned to the maternal rectal microbiome. Substantial microbiota transfer from the maternal rectum (and decreased transfer from the cervix) to neonatal stool and ear-skin samples was identified, especially when comparing ear-skin samples of vaginally delivered neonates of mothers with type 1 diabetes with vaginally delivered neonates born to control mothers. In total, 83.2±19.8% of the ear-skin microbiome in neonates born to mothers with type 1 diabetes and 59.5±31.1% of the ear-skin microbiome in neonates born to control mothers was attributed to maternal microbiota of the rectum, and 0.7±1.1% of the ear-skin microbiome in neonates born to mothers with type 1 diabetes and 5.2±9.2% of the ear-skin microbiome in neonates born to control mothers was attributed to maternal microbiota of the cervix. These findings were limited to vaginally born neonates.

## Discussion

Although the impact of maternal GDM, maternal obesity, delivery mode and intrapartum antibiotics on neonatal microbiomes and neonatal health has been evaluated [12–14, 35–39], reports of microbiome characteristics in type 1 diabetes are limited. In the ENDIA study an important difference in gut microbiome composition (based on beta diversity indices) between gravidae with type 1 diabetes and control gravidae in the third trimester of pregnancy has been found [8]. In addition, in the TEDDY and ABIS studies, some gut bacteria were found to precede seroconversion and diagnosis of type 1 diabetes [17, 20]. In another study of eight women, an association of a ‘prevalent’ maternal vaginal microbiome (measured up to 11 years after pregnancy) with the occurrence of type 1 diabetes in their offspring was demonstrated [22]. In our study, an influence of type 1 diabetes on the overall microbial composition was revealed in maternal rectum swabs and neonatal stool samples only, and in a more detailed analysis we identified the microbiota associated with the disease. In the TEDDY study, five bacterial genera were associated with type 1 diabetes onset (development of islet autoimmunity) in early childhood, with *Parabacteroides* the most statistically significant (*p*<0.001). Moreover, 16 bacterial genera were less abundant in children with type 1 diabetes, including five unclassified genera of the family *Ruminococcaceae* and *Lactococcus*, *Streptococcus* and *Akkermansia* [16]. In our study, *Parabacteroides* was also found to be over-represented (in the cervix; *p*<0.001) while *Streptococcus* was under-represented (in the vaginal canal; *p*<0.001) in samples derived from women with type 1 diabetes. Although the presence of these pathogenic genera remains to be further elucidated, this finding might not be incidental. For example, *Sneathia*, which in our study was over-represented in the rectum swabs, vaginal canal and cervix of women with type 1 diabetes, has been characterised as an emerging pathogen (*Sneathia amnii*) that may affect pregnancy outcomes [40]. Importantly, in our study, seven of the microbiota genera specific for women with type 1 diabetes were found in their neonates. These findings were not attributable to maternal weight or parity.

To further explore the effect of glycaemic control on microbiota composition, we investigated the effects of maternal HbA_1c_ levels. HbA_1c_ levels measured just before delivery did not influence the microbiota composition (maternal or neonatal); however, glycaemic dysregulation before pregnancy (represented by HbA_1c_ levels in the first trimester of pregnancy) may influence offspring microbiota community composition in the long term. Based on our results, we assume that the maternal microbiomes in women with type 1 diabetes contributed, to an unknown extent, to the establishment of the neonatal microbiomes in utero.

There are a growing number of reports suggesting that Caesarean delivery per se is not associated with any appreciable differences in neonatal microbiota compared with vaginal delivery [36, 41–45]. Here, in line with studies reporting that there is an association [35, 36, 38, 39], we confirmed an influence of delivery mode on microbiota composition in the neonatal samples assessed.

Previously, no differences were found between the microbiota composition of neonates within 24 h of the administration of antibiotics (cephalosporins) to mothers as Caesarean section prophylaxis and the microbiota composition of neonates born vaginally without intrapartum antibiotics [46]. However, in prospective studies, an impact of intrapartum antibiotics on infant gut microbiota composition and maturation was reported, characterised by lower abundances of *Bacteroides* after penicillin administration and *Bifidobacterium* after cephalosporin administration, as well as enrichment of *Veillonella dispar* after multi-drug intervention [37]. Here, no statistically significant differences were found between the women with type 1 diabetes and control women in the number of antibiotics administered and the percentage receiving antibiotic prophylaxis, but prophylaxis itself influenced the composition of the neonatal microflora, regardless of maternal type 1 diabetes status.

Little is known about the effects of maternal diet during pregnancy on maternal/neonatal microbiota. Information on dietary aspects in the gravidae with type 1 diabetes examined in this study is discussed in detail elsewhere [32]. Women with type 1 diabetes consumed less carbohydrate and more protein than control women, and the percentage of energy derived from carbohydrates was decreased in their diet while the percentage of energy derived from proteins was increased. These results appear to be related to the differences in microbiota composition between the two groups of pregnant women assessed. Concerning the diets of gravidae and prophylactic supplementation, in line with the results of the ENDIA study [8], we found that *Bifidobacterium* was under-represented in samples derived from women with type 1 diabetes. Our subsequent analysis indicated that consumption of probiotics with *Bifidobacterium* may influence the neonatal microbiome composition, but further studies are needed to verify this finding.

Vertical transmission of microbiomes from mothers to fetuses [43], as well as direct bacterial transfer from mothers to infants [16], have been investigated previously. In our study, substantial microbiota transfer from the maternal rectum to neonatal samples was identified. In contrast to a previous study [41], we found an increased contribution of maternal microbiota (rectum) to neonates (stool samples) in those born by Caesarean section compared with those born vaginally. In the study by Bogaert et al, covering the 30 first days of life, on average, 58.5% of the infant microbiota was found to be attributed to maternal sources [41]. Here, we found that, on average, 81% of neonatal microbiota can be attributed to maternal sources, with a predominance of the rectal microbiome (69%). No substantial relationship was found when assessing the transfer of microbiota from the maternal reproductive tract to neonates. In summary, therefore, the transfer of microbiota from the maternal reproductive tract to neonates was limited and from the maternal rectum to neonates was substantial, and in both cases was specific for each maternal–neonatal dyad. In addition, in a previous study, overlap between maternal vaginal microbiota and infant faecal microbiota was found to be minimal, in contrast to overlap with maternal rectal microbiota [47]. Ferretti et al also showed that maternal gut microbiota were more persistent in infants’ gut and ecologically better adapted than bacteria acquired from other sources [38].

### Study strengths

We designed the study so that all biological samples were collected from the same hospital and wards and, moreover, the same clinical staff collected and processed all of the samples. Moreover, as ethnic origin is important in the context of microbiomes [48], in this study we included participants with the same ethnicity. We comprehensively analysed four maternal sampling sites and two at-birth neonatal sampling sites, taking into account detailed clinical metadata and paired mother-to-neonate microbial transfer.

### Study weaknesses

A weakness of this study is that only selected maternal and neonatal microbiomes were evaluated. In our study we did not include positive mock controls or samples from the hospital environment; however, we did assess negative controls in each molecular experiment. The experimental reagents were certified for low bioburden and have been validated for bias-free microbial DNA extraction (by the manufacturer, Zymo Research). As the outcomes of metagenomics studies are not very quantifiable (cut-off points for the interpretation of basic clinical research data have not been established) and, moreover, some factors influencing the microflora in microbiomes remain unidentified, we could not further interpret the results obtained.

### Conclusion

In summary, in this study we characterised various microbiomes and identified the microbiota specific for type 1 diabetes in both mothers and their neonates. Our study provides evidence for the influence of maternal type 1 diabetes on neonatal microbiomes. Although the status of type 1 diabetes cannot be changed, our results provide further arguments in favour of normalising HbA_1c_ and BMI levels when planning a pregnancy. Subsequent longitudinal studies of neonates born to mothers with type 1 diabetes should be conducted to further characterise the stability of neonatal colonisation by microbiota associated with maternal disease.

### Supplementary Information

Below is the link to the electronic supplementary material.

Supplementary file1 (PDF 2.40 MB)

## Data Availability

The BioProject (PRJNA961636) and associated SRA metadata are available at http://www.ncbi.nlm.nih.gov/bioproject/961636. Processed data on probiotic supplementation and the PICRUSt analysis are available in the Mendeley Data Repository (https://doi.org/10.17632/g68rwnnrfk.1).

## Abbreviations

ABIS: All Babies In Southeast Sweden
AGA: Appropriate for gestational age
ASV: Amplicon sequence variants
ENDIA: Environmental Determinants of Islet Autoimmunity
GDM: Gestational diabetes mellitus
HSD: Honestly significant difference
LGA: Large for gestational age
PCoA: Principal coordinates analysis
PICRUSt: Phylogenetic Investigation of Communities by Reconstruction of Unobserved States
SGA: Small for gestational age
TEDDY: The Environmental Determinants of Diabetes in the Young
