# Supplementary material for: Effects of maternal type 1 diabetes and confounding factors on neonatal microbiomes
Source: Diabetologia. 2023 Nov 29;67(2):312–26. doi: 10.1007/s00125-023-06047-7 (PMC10789840; doi:10.1007/s00125-023-06047-7)
Supplement: Supplementary file 1 — Supplementary file1 (PDF 2.40 MB) [file 125_2023_6047_MOESM1_ESM.pdf]

ELECTRONIC SUPPLEMENTARY MATERIALS

Effects of maternal type 1 diabetes and confounding factors on neonatal microbiomes

Marzena Gajecka[1,2], Pawel Gutaj[3], Katarzyna Jaskiewicz[2], Malgorzata Rydzanicz[4], Tomasz Szczapa[5], Dorota Kaminska[1], Grzegorz Kosewski[6], Juliusz Przyslawski[6], Rafal Ploski[4], Ewa Wender-Ozegowska[3]

[1]Chair and Department of Genetics and Pharmaceutical Microbiology, Poznan University of Medical Sciences, Poznan, Poland
[2]Institute of Human Genetics, Polish Academy of Sciences, Poznan, Poland
[3]Department of Reproduction, Poznan University of Medical Sciences, Poznan, Poland
[4]Department of Medical Genetics, Medical University of Warsaw, Warsaw, Poland
[5]Department of Neonatology, Poznan University of Medical Sciences, Poznan, Poland
[6]Chair and Department of Bromatology, Poznan University of Medical Sciences, Poznan, Poland

Marzena Gajecka, Pawel Gutaj and Katarzyna Jaskiewicz contributed equally to this study

ORCID iDs
Marzena Gajecka https://orcid.org/0000-0001-7468-2604
Pawel Gutaj https://orcid.org/0000-0003-2885-9792
Katarzyna Jaskiewicz https://orcid.org/0000-0002-6380-5119
Malgorzata Rydzanicz https://orcid.org/0000-0002-6969-0535
Tomasz Szczapa https://orcid.org/0000-0002-5214-2719
Dorota Kaminska https://orcid.org/0000-0001-5934-1115
Grzegorz Kosewski https://orcid.org/0000-0002-6380-7704
Juliusz Przyslawski https://orcid.org/0000-0001-9205-2817
Rafal Ploski https://orcid.org/0000-0001-6286-5526
Ewa Wender-Ozegowska https://orcid.org/0000-0002-5492-8651

Corresponding author:
Marzena Gajecka,
email gamar@man.poznan.pl

Business Standard

1. ESM Materials and Methods

1.1. Participant ascertainment

Gravidae with type 1 diabetes, control gravidae without type 1 diabetes, and their neonates were ascertained in the Gynecologic and Obstetrical University Hospital at the Poznan University of Medical Sciences, Poznan, Poland, in accordance with the ascertainment schemes presented in ESM Figure 1. The Department, including its outpatient clinic, is the biggest perinatal center for gravidae with diabetes in Poland. It provides care for patients from the Greater Poland voivodeship, with a population of approximately 3.4 million. As the aspect of ethnic origin is important in the context of microbiomes' differences, and due to limited funding, we decided to study participants with the same ethnicity (ethnicity was self-declared by participants). The process of care delivered to women with type 1 diabetes without complications was based on at least three planned visits during pregnancy: in the first trimester, in mid-pregnancy (20-24 weeks of gestation), and near delivery (34-39 weeks of gestation). Participants who required more vigilant surveillance were admitted more frequently. In the meantime, patients were referred biweekly for regular check-ups in the hospital-based outpatient clinic and also received regular outpatient consultations with a specialist in diabetology. Hospitalized type 1 diabetes participants also had regular consultations with certified Diabetes Nurse Educators focusing on proper insulin therapy, glycemic control, and dietary education.

Inclusion criteria. According to national guidelines [1], all women from the control group were screened for hyperglycemia in pregnancy and were free of diabetes mellitus in pregnancy or gestational diabetes mellitus (GDM). Screening protocol in Poland starts in the first trimester and consists of either fasting blood glucose measurement in women without risk factors for GDM or the 75 grams oral glucose tolerance test (75 g OGTT) in women with risk factors for GDM. The risk factors are as follows: pregnancy beyond 35 years of age; history of macrosomia (birth weight > 4000 g); previous delivery of a neonate with a congenital anomaly; history of intrauterine fetal demise; hypertension; overweight and obesity; family history of diabetes type 2; GDM during previous pregnancies; multiparity; polycystic ovary syndrome. If screened negative in the first trimester (fasting blood glucose <5.1 mmol/L/normal 75g OGTT) every woman is referred for the 75g OGTT between 24-28 weeks of gestation. Thresholds in the 75 g OGTT used for screening

Business Standard

in Poland are based on the Hyperglycemia and Adverse Pregnancy Outcomes study (HAPO) results [2], and are in line with IADPSG [3] and WHO [4] recommendations. The result of the 3-step 75 g OGTT is considered normal when the fasting level is <5.1 mmol/L; 60 minutes level <10 mmol/L; and 120 minutes level <8.5 mmol/L.

HbA_{1c} levels assessment. The gravidae' blood samples were taken after overnight fasting and proceeded in the central laboratory of the Gynecologic Obstetrical University Hospital in Poznan for analysis. The HbA_{1c} levels were determined using the turbidimetric inhibition immunoassay TINIA (Tina-quant Hemoglobin A1c II test in a Cobas c311 analyzer; Roche Diagnostics, Basel, Switzerland). The total serum cholesterol, HDL cholesterol, and triglyceride (TG) levels were determined with Cholesterol CHOD-PAP (Roche Diagnostics), HDL-C plus (Roche Diagnostics), and Triglycerides GPO-PAP (Roche Diagnostics), respectively, on a Cobas c501 analyzer (Roche Diagnostics).

Nutritional assessment. Gravidae with type 1 diabetes participated in a dietary education program led by diabetes educators during pregnancy. The control group women did not receive any nutritional counseling either in early pregnancy or during term hospitalization. The ascertained women underwent detailed nutritional assessment, using 24-hour dietary recall for seven days. Moreover, information about the participants was collected based on the author's survey (ESM Participant Survey is provided below) and the assessment of the validated Food Frequency Questionnaire FFQ-D10 [5]. Detailed protocols of nutritional assessment and dietary habits, as well as study results, were discussed in detail elsewhere [6]. Briefly, it was carried out face-to-face, using 24-hour dietary recalls for 7 consecutive days (day-by-day) before delivery. The Dietetyk 2011 computer software (JuMaR, Poznan, Poland) based on a Polish database comprising tables of the nutritional value of food [7] was used to perform a qualitative and quantitative analysis of daily food intake. Women were asked to indicate the frequency of consumption of foodstuffs within the last 12 months with a special focus on the period of their pregnancy. The results were converted to a daily frequency indicating how many portions of a selected product were consumed within one day [6].

1.2. 16S rRNA gene sequencing and data analysis

16S rRNA gene sequencing method was adapted from the methods developed by Weisburg [8]. The genomic DNA was extracted using the ZymoBiomics DNA Miniprep Kit (Zymo

Business Standard

Research, USA) following the manufacturer's instructions. Briefly, samples (swabs or up to 250 mg of the stool samples) were placed in a ZR BashingBead™ Lysis Tubes and lysed with ZymoBIOMICS™ Lysis Solution using shaking block (20min, 2500rpm, Eppendorf Thermomixer, Germany). Next, the supernatant was transferred to the Zymo-Spin™ III-F filter, and after centrifuging and adding the ZymoBIOMICS™ DNA Binding Buffer, the mixture was placed at Zymo-Spin™ IIC-Z Column. The column was three times washed and the DNA was eluted with DNase/RNase Free Water. The V3-V4 region of the 16S rRNA gene was amplified by PCR (16S Amplicon PCR Forward Primer = 5' TCGTCGGCAGCGTCAGATGTGTATAAGAGACAGCCTACGGGNGG CWGCAG, and 16S Amplicon PCR Reverse Primer = 5' GTCTCGTGGGCTC GGAGATGTGTATAAGAGACAGGACTACHVGGGTATCTAATCC) and sequenced on the MiSeq platform (Illumina, USA) in Department of Medical Genetics at Medical University of Warsaw, using the 2×250 bp paired-end read protocol. Negative controls were included in each PCR experiment (performed to generate 16S rRNA gene amplicons) to confirm the microbiological purity of the experimental environment and applied reagents.

16S rRNA sequencing data analysis. Raw 16S rRNA gene sequence data were processed using DADA2 [9] pipeline (v1.6) in R (v3.4.4). Sequences were examined for sequencing quality and trimmed. Amplicon Sequence Variants (ASVs) were identified per sample after sequence de-replication. Chimeric ASVs were removed using the function removeBimeraDenovo. Next, ASVs were assigned using the RDP classifier [10] against the SILVA database (v138) [11]. Read counts for each ASV and taxonomy of the ASVs were tabulated for downstream analysis in the R package phyloseq (v1.22.3) [12].

For beta diversity measurements PCoA plot comparisons of maternal and neonate microbiome community composition by disease state across vaginal introitus, vaginal canal in the middle, cervix, and rectum as well as neonatal sample types were performed. PCoA plot coordinates are based on Weighted UniFrac dissimilarity indices obtained from ASV counts transformed using variance stabilizing transformation (VST). Trendlines are derived from fitting generalized linear models for each factor and statistical significance annotations are based on adonis2 permutational analysis of variance (PERMANOVA) scores (permutations = 9999).

Business Standard

Heatmaps were generated using the R package pheatmap (v1.0.12) [13].

Hierarchical clustering was performed using complete linkage on Bray-Curtis dissimilarity distances using the R package vegan (v2.5-7) [14] as indicated.

Bacteria associated with type 1 diabetes in the mothers (differentially expressed bacterial genera) were calculated using the Wald test with Benjamini and Hochberg correction for multiple testing, based on bacterial relative abundance data.

Neonatal microbiota profiles. Microbiome profiles of neonatal ear-skin swabs or stool samples were established and presented as the heat-tree comparisons of the log2 median ratios in the neonatal microbiome profiles of ear-skin swabs or stool samples, assessed by type 1 diabetes disease state.

PICRUSt Analysis. For a prediction of metagenome function, we used Phylogenetic Investigation of Communities by Reconstruction of Unobserved States (PICRUSt2, v.2.3.0 beta) with default parameters [15].

Effect of glycemic control on microbiota composition. To assess the effect of long-term glycemic control on microbiota composition we compared the relative abundance of all genera based on ASVs and taxonomy tables taking into account the HbA_{1c} levels in the first, second, and third trimesters. Microbiota community composition in subgroups of type 1 diabetes women with $HbA_{1c} \leq 55$ mmol/mol (≤7.2%) vs. $HbA_{1c} > 55$ mmol/mol (>55 mmol/mol was chosen as discriminative value for sensitivity analysis), as measured in the first trimester of pregnancy, across vaginal introitus, vaginal canal in the middle, cervix and rectum as well as neonatal sample types was assessed. The obtained PCoA plots coordinates are based on Weighted UniFrac dissimilarity indices obtained from VST transformed ASV counts. Trendlines are derived from fitting generalized linear models for each factor and statistical significance annotations are based on adonis2 PERMANOVA scores (permutations = 9999). Missing first trimester HbA_{1c} values were either imputed using a KNN (k-Nearest-Neighbours) based model from the remaining metadata values for participants with type 1 diabetes or generated based on a norm distribution derived from average first trimester HbA_{1c} values for the mothers (31 mmol/mol; 4.96% ± 0.53%). Graphical representations and associations with type 1 diabetes /delivery mode were performed with phyloseq (v1.22.3) [12], ggpubr (v0.4.0) [16], and matrixTests (v 0.1.9.1)

Business Standard

[17] R packages, and JASP (v0.16) [18]. Up- and downregulated metabolic pathways were identified/interpreted by referring to the MetaCyc database [19].

2. ESM Results

2.1. Clinical characteristics of maternal-neonatal dyads

Indications for caesarean sections. Indications for caesarean sections in women with type 1 diabetes were as follows: suspicion of LGA on ultrasound (*n*=12), diabetic retinopathy (*n*=9), long-lasting diabetes (*n*=4), previous caesarean section (*n*=2), failed induction of labour (*n*=3), orthopedic (*n*=1), and epilepsy (*n*=1). Indications for cesaream section in women in the control group were as follows: previous caesarean section (*n*=6), caesarean section on demand (*n*=5), breech presentation (*n*=3), suspicion of LGA on ultrasound (*n*=1), failed induction of labour (*n*=2), previous myomectomy (*n*=1), previous IIIrd degree perineal rupture (*n*=1), and orthopedic (*n*=1).

2.2. Maternal microbiome community composition and bacteria associated with type 1 diabetes

Diversity of bacteria in the three assessed vaginal sampling sites. The differences in phylum relative abundance between the three assessed vaginal sampling sites were found. Firmicutes, Proteobacteria, Actinobacteria, and Bacteroidetes dominated among the 12 examined phyla. The low variability of bacteria near the cervix and the ascendent trend of phylum Firmicutes, increasing from introitus through the center of the vagina to the cervix were found in both type 1 diabetes and control women (Figure 1; introitus vs. cervix: p=0.087). Proteobacteria dominated the introitus of the vagina of both women with type 1 diabetes and control women compared to other sampling sites (p=0.001), and were almost absent in the cervix, while the relative abundance of Actinobacteria and Bacteroidetes were comparable (p>0.05) in the assessed vaginal sampling sites (Figure 1).

In contrast to previously reported microbiota homogeneity in three vaginal sampling sites among gravidae [20], we observed remarkable vaginal niche variability in the relative abundance. This is consistent with prior findings in non-gravid subjects [21, 22] and gravidae, where decreased diversity and richness of the vaginal microbiome in upper vaginal sites including in the posterior fornix was demonstrated [23–25]. In the current study we further contribute to this topic and show that variation in both richness and

Business Standard

diversity is also observed in the cervical subsite. Generally, the low variability of bacteria near the cervix and the ascendent trend of phylum Firmicutes, increasing from introitus through the center of the vagina to the cervix were revealed in both women with type 1 diabetes and controls. Previously, vaginal species in 34 Chinese women during different pregnancy stages were characterized at the cervix, posterior fornix, and vaginal canal, using the Illumina sequencing of 16S rRNA tag sequences [20]. Little heterogeneity across community structures within each individual was reported, as determined by LEfSe, indicating high vaginal microbiome homogeneity at the examined vaginal sites [20].

2.3. Differences in microbiota composition caused by confounding variables

Impact of antibiotic treatment on bacterial diversity. There were no statistically significant differences in numbers of antibiotic prophylaxis between the studied groups ($p>0.05$; Table 1). Caesarean section prophylaxis, Streptococcus agalactiae colonization, and premature rupture of membranes prophylaxis were the causes of antibiotic treatment in both women with type 1 diabetes and controls (Table 1). Cefuroxime and cefazolin were the most frequently administrated antibiotics in women with type 1 diabetes (51.5% and 31.5.%, respectively) and control women (30.5% and 43.5%, respectively) (Table 1).

Dietary aspects. The intake of probiotics during pregnancy in the form of probiotics and/or synbiotics (the qualitative data) is summarized in ESM Table 1. Since pregnant women took different types of supplements during pregnancy, and the supplements contained a different number of bacterial strains, it was impossible to accurately determine the amounts of probiotics taken.

The percentage of energy derived from protein, estimated based on 24-hour dietary recall for 7-days, received from 33 gravidae with type 1 diabetes and 14 controls (ESM Figure 9), influenced maternal and neonatal microbiota composition, regardless of the type 1 diabetes disease status ($p=0.002$)(ESM Table 6). Also, in the multivariate analysis, the disease and energy derived from proteins influenced the microbiota composition ($p=0.003$)(ESM Table 6). Previously, the decrease of protein consumption has been reported to correlate with the increase of Bacteroides in one-year-old children with type 1 diabetes [26], whereas in our study a statistically significant decrease in abundance of Bacteroides in stool samples of neonates born to mothers with type 1 diabetes was found.

Business Standard

Microbial functional profiles dysregulated in type 1 diabetes. The PICRUSt analysis of maternal and neonatal samples (evaluated together) in the aspect of maternal type 1 diabetes status pointed to 18 altered pathways involved in microbiota metabolism. Further evaluating these results, 6 pathways were predicted as enriched in the samples derived from the control women and their neonates, namely pathways of lactose and galactose degradation (class: Carbohydrate Degradation), 6-hydroxymethyl-dihydropterin diphosphate biosynthesis I (class: Folate Biosynthesis), acetyl-CoA fermentation to butanoate II (class: Generation of Precursor Metabolites and Energy), superpathway of UDP-N-acetylglucosamine-derived O-antigen building blocks (class: Carbohydrate Biosynthesis), aromatic biogenic amine degradation (bacteria) (class: Amine and Polyamine Degradation), and S-methyl-5-thio-α-D-ribose 1-phosphate degradation (class: Nucleoside and Nucleotide Degradation) (ESM Figure 10).

In the gravidae rectal swabs, 10 pathways were predicted including 2 pathways from the carbohydrate biosynthesis class. In women with type 1 diabetes decreased folate biosynthesis (N10-formyl-tetrahydrofolate biosynthesis) was noticed. The SCFA production in the rectal swabs of women with type 1 diabetes was found lowered (acetyl-CoA fermentation to butanoate II, purine nucleobases degradation I (anaerobic)). The Generation of Precursor Metabolites and Energy (from superpathway of glycolysis and Entner-Doudoroff) was also decreased.

Based on microbiota data obtained from three vaginal sampling sites, it was noticed that 165 pathways were notably differentiated when comparing samples from women with type 1 diabetes with samples from control gravidae. Pathways belonging to the Amino Acid Biosynthesis node were overrepresented in type 1 diabetes. Predicted lactate production as an indirect product of selected Carbohydrate Degradation pathways (superpathway of glucose and xylose degradation, fucose degradation, L-rhamnose degradation I) was remarkably enriched in samples of women with type 1 diabetes, but as a product of selected Fermentation pathways (pyruvate fermentation to acetate and lactate II, homolactic fermentation) was enriched in samples derived from control gravidae. For more details of functional profiling please see Mendeley Data Repository (10.17632/g68rwnnrfk.1).

The effect of the gut microflora on the host is a complex interaction, mainly based on the products of bacterial metabolism that affect the host’s metabolism [27], intestinal barrier

Business Standard

integrity [28], and immunity [29]. However, since multiple taxa have overlapping metabolic functions, subtle variations in which microbiota occupy a niche may not contribute to physiologically meaningful differences in the host [30]. Still, the influence of the microbiota of the female reproductive tract can be considered in relation to the influence of the products of bacterial metabolism on the course of pregnancy, especially in pregnancy with type 1 diabetes, in which numerous complications arise [31].

In this study, type 1 diabetes disease status seems to cause substantial distortion among others in pathways of Carbohydrate Degradation/Biosynthesis and Folate Biosynthesis. In the gravidae rectal swabs decreased carbohydrate, folate, and SCFA biosynthesis (N10-formyl-tetrahydrofolate biosynthesis) was noticed, what is in line with the previous findings describing the greatest functional change of gut microbiota (increased production of lipopolysaccharides and decreased production of folates and SCFA) in the third trimester of pregnancy in gravidae with type 1 diabetes [32]. Also, LPS biosynthesis pathways (O-antigen building blocks biosynthesis, superpathway of UDP-N-acetylglucosamine-derived O-antigen building blocks biosynthesis), which seemed to take part in a potent innate immune activation [33], were predicted to be less represented in gravidae with type 1 diabetes. All these changes could contribute to more pro-inflammatory gut profile and next increased risk of pregnancy complications.

Interestingly, pathways belonging to Amino Acid Biosynthesis node were increased in type 1 diabetes with no exception. Moreover, we found enhanced branched amino-acids biosynthesis in reproductive tract samples of type 1 diabetes mothers (L-isoleucine biosynthesis I (from threonine), superpathway of branched amino acid biosynthesis, L-isoleucine biosynthesis II, L-isoleucine biosynthesis III), what was already discovered in gut's obese type 2 diabetes individuals [34]. Among 15 of the most differentiated pathways, four are responsible for vitamin K2 biosynthesis (superpathway of menaquinol-6 biosynthesis I, superpathway of menaquinol-8 biosynthesis I, superpathway of menaquinol-9 biosynthesis, superpathway of menaquinol-10 biosynthesis) which was suggested to improve insulin sensitivity within type 2 diabetes individuals after oral supplementation [35], however the biological effect in type 1 diabetes and genital tract remain unascertained. Still, in type 1 diabetes reproductive tract functional metagenomics data we found some pathways whose products were previously reported to be enriched in

Business Standard

bacterial vaginosis, such as aspartate superpathway [36]. Moreover, some pathways (superpathway of glycolysis, pyruvate dehydrogenase, TCA, and glyoxylate bypass, (5Z)-dodec-5-enoate biosynthesis, pyrimidine deoxyribonucleotides de novo biosynthesis II) overrepresented in type 1 diabetes were reported enriched in vaginal samples from individuals with primary ovarian failure [37].

Breastfeeding. 14% and 95% of the neonates were exclusively breastfed, by women with type 1 diabetes and control women, respectively (P<0.001). Mixed feeding, including mother’s milk and formula milk, was administered to 82% of neonates of mothers with type 1 diabetes (Table 2).

In the TEDDY study [38], breastfeeding was the only covariate that was importantly associated with microbial metabolic potential, however regardless of whether it was exclusive or together with formula milk and/or solids. In another study, the delivery mode and feeding mode were found as the main covariates shaping the dissimilarity (Bray-Curtis distances) between newborns’ stool microbiota at day 3 [39]. The C-section delivery and mixed feeding resulted in decrease of *Bifidobacterium* species relative abundances, and the absence of *Bacteroides,* in neonatal stool microbiota at day 3 [39]. However, as our study showed substantial differences in the numbers of breastfed and mixed breastmilk/formula fed neonates, comparing type 1 diabetes vs. control mothers, the influence of the way the neonates were fed on microbial diversity was not further investigated in the subgroups of feeding type.

Business Standard

3. **ESM Tables**

ESM Table 1. Clinical characteristics of women with type 1 diabetes (T1D) and control women.

Clinical characteristic	T1D group (*n*=50)	Control group (*n*=42)	*p* value
Age (year), mean ± SD	30.5 ± 4.1	32.4 ± 3.7	0.011
Age at delivery (full years), mean ± SD	30 ± 4	32 ± 4	0.008
Ethnicity, White[a]	50 (100.0)	42 (100.0)	
Geographical location, Poland	50 (100.0)	42 (100.0)	
Delivery mode			
Caesarean	33 (66.0)	20 (47.6)	0.118
Unscheduled	2 (4.0)	2 (4.8)	
Scheduled	31 (62.0)	18 (42.9)	
Vaginal delivery	12 (24.0)	17 (40.5)	
Vacuum-assisted vaginal delivery	5 (10.0)	5 (11.9)	
Prior C-sections			
Once	7 (14.0)	5 (11.9)	0.736
Twice	1 (2.0)	2 (4.8)	
None	42 (84.0)	35 (83.3)	
Preterm (<37 weeks)			
Yes	0 (0.0)	0 (0.0)	
No	50 (100.0)[b]	42 (100.0)	
Gestation (weeks), mean ± SD	38.1 ± 0.7	39.2 ± 1.0	<0.001
Parity			
Primiparity	28 (56.0)	14 (33.3)	0.038
Multiparity	22 (44.0)	27 (64.3)	
NA	0 (0.0)	1 (2.4)	
Miscarriages			
Yes	7 (14.0)	8 (19.1)	0.751
No	41 (82.0)	33 (78.6)	
NA	2 (4.0)	1 (2.4)	
Pre-pregnancy BMI (kg/m^2), mean ± SD[c]	23.19 ± 2.95	22.46 ± 2.56	0.423
Pre-pregnancy BMI[c]			
Underweight	1 (2.0)	1 (2.4)	0.561
Normal	35 (70.0)	33 (78.6)	
Overweight	13 (26.0)	7 (16.7)	
Obese	1 (2.0)	0 (0.0)	
NA	0 (0.0)	1 (2.4)	
Before-delivery BMI (kg/m^2), mean ± SD[c]	28.12 ± 3.75	27.46 ± 2.97	0.649
Weight gain during pregnancy (kg), mean ± SD[c]	13.61 ± 5.34	14.00 ± 3.79	0.727
Weight gain[d]			
Non-excessive	32 (64.0)	26 (61.9)	0.954
Excessive	18 (38.0)	15 (35.7)	

Business Standard

NA	0 (0.0)	1 (2.4)	
Energy derived from fat [%][e], mean ± SD	30.80 ± 5.09	27.79± 4.83	0.051
Energy from saturated fatty acids [%][e], mean ± SD	12.97 ± 2.46	11.69 ± 2.59	0.075
Energy derived from carbohydrates [%][e], mean ± SD	50.85 ± 5.33	55.47 ± 5.30	0.017
Energy derived from total protein [%][e], mean ± SD	18.35 ± 3.01	16.74 ± 1.59	0.030
HbA_{1c} levels met targets during pregnancy[f]			
Yes	50 (100.0)	0 (0.0)	-
No	0 (0.0)	42 (100.0)	-
Maternal diabetes			
T1D	50 (100.0)	0 (0.0)	-
T2D	0 (0.0)	0 (0.0)	-
Gestational	0 (0.0)	0 (0.0)	-
None	0 (0.0)	42 (100.0)	-
Age at T1D diagnosis, mean ± SD	17.91 ± 8.21	NA	-
Duration of T1D (years), mean ± SD	12.56 ± 7.02	NA	-
Diabetes medication (insulin therapy)			
Yes	50 (100.0)	0 (0.0)	-
No	0 (0.0)	42 (100.0)	-
Time on insulin therapy (years), mean ± SD	12.52 ± 17.09	NA	-
Age at the beginning of insulin therapy (years), mean ± SD	17.97 ± 8.30	NA	-
Classification of pregestational diabetes (modified P. White classification)[d]			
Class A	0 (0.0)	NA	-
Class B	18 (36.0)	NA	-
Class C	15 (30.0)	NA	-
Class D	11 (22.0)	NA	-
Class R	5 (10.0)	NA	-
Class F	0 (0.0)	NA	-
Class RF	1 (2.0)	NA	-
Class H	0 (0.0)	NA	-
Class T	0 (0.0)	NA	-
Non-proliferative Retinopathy			
Yes	5 (10.0)	0 (0.0)	0.040
No	45 (90.0)	42 (100.0)	
Proliferative Retinopathy			
Yes	7 (14.0)	0 (0.0)	0.015
No	43 (86.0)	42 (100.0)	
Retinopathy			
Yes	12 (24.0)	0 (0.0)	0.002
No	38 (76.0)	42 (100.0)	
Hypothyroidism			
Yes	22 (44.0)	10 (23.5)	0.102
No	28 (56.0)	32 (76.5)	
Antibiotic prophylaxis			
Yes	34 (68.0)	20 (47.6)	0.048
No	16 (32.0)	22 (52.4)	
Cause of antibiotics prophylaxis[g]			

Caesarean section prophylaxis	28 (80.0)	12 (54.5)	0.083
Streptococcus agalactiae	5 (14.3)	5 (22.7)	
Chorioamnionitis (intra-amniotic infection)	0 (0.0)	0 (0.0)	
Maternal bacterial endocarditis prophylaxis	0 (0.0)	0 (0.0)	
Premature rupture of membranes prophylaxis	2 (5.7)	5 (22.7)	
Used Antibiotic[h]			
Cefuroxime	18 (51.4)	7 (30.4)	0.385
Ampicillin	6 (17.1)	4 (17.4)	
Cefazolin	11 (31.4)	10 (43.5)	
Clindamycin	0 (0.0)	1 (4.3)	
Cefalexin	0 (0.0)	1 (4.3)	
Any chronic disease/disorder (excluding T1D)[i]			
Yes	6 (12.0)	1 (2.4)	<0.001
No	44 (88.0)	41 (97.6)	
Hypertension			
Yes	3 (6.0)	0 (0.0)	0.112
No	47 (94.0)	42 (100.0)	
Gestational hypertension			
Yes	2 (4.0)	1 (2.4)	0.663
No	48 (96.0)	41 (97.6)	
Maternal preeclampsia			
Yes	2 (4.0)	0 (0.0)	0.195
No	48 (96.0)	42 (100.0)	
Supplements _probiotics before pregnancy (excluding synbiotics)			
Yes	8 (16.0)	3 (7.1)	0.333
No	32 (64.0)	27 (64.3)	
NA	10 (20.0)	12 (28.6)	
Supplements _probiotics during pregnancy (excluding synbiotics)			
Yes	4 (8.0)	6 (14.3)	0.316
No	36 (72.0)	24 (57.1)	0.379
NA	10 (20.0)	12 (28.6)	0.402
Supplements _synbiotics before pregnancy			
Yes	7 (14.0)	13 (31.0)	0.040
No	33 (66.0)	17 (40.5)	
NA	10 (20.0)	12 (28.6)	
Supplements _synbiotics during pregnancy			
Yes	2 (4)	17 (40)	<0.001
No	38 (76)	13 (31)	
NA	10 (20)	12 (29)	
Supplements _probiotics before pregnancy (including synbiotics)			
Yes	12 (24.0)	15 (35.7)	0.151
No	28 (56.0)	15 (35.7)	
NA	10 (20.0)	12 (28.6)	
Supplements _probiotics during pregnancy (including synbiotics)			
Yes	5 (10.0)	29 (69.0)	<0.001
No	35 (70.0)	1 (2.4)	

NA	10 (20.0)	12 (28.6)	
Probiotics in food before pregnancy[j]			
Yes	39 (78.0)	27 (64.3)	0.265
No	1 (2.0)	3 (7.1)	
NA	10 (20.0)	12 (28.6)	
Probiotics in food during pregnancy[j]			
Yes	39 (78.0)	30 (71.4)	0.365
No	1 (2.0)	0 (0.0)	
NA	10 (20.0)	12 (28.6)	
Processed samples[k]	50 (100.0)	41 (97.6)	

Data are *n* (%) unless reported otherwise

Statistical significance is based on the two-tailed χ^2 test and two-tailed Mann–Whitney *U* test for qualitative and quantitative data, respectively

[a]Self-reported the participant

[b]One delivery at 36 weeks and 6 days of pregnancy

[c]Weight before pregnancy was self-reported and BMI was calculated by a researcher; weight and BMI just before delivery were measured and calculated in the hospital

[d]According to Wender-Ożegowska et al. [40]

[e]Counting based on 24-hours dietetic recall for 7-days received from 33 T1D participants and 14 control participants

[f]HbA_{1c} measurement in each of the three trimesters of pregnancy

[g]In one T1D participant and two control participants there was more than one cause of using the antibiotics profilaxy

[h]In one T1D participant and three control participants more than one antibiotic was administrated

[i]Hypertension, asthma, epilepsy

[j]Yoghurts, kefirs, acidophilic milk, bifidus milk, curdled milk, buttermilk, probiotic drinks, cottage cheese, rennet cheese, blue cheese, probiotic bran, pickled products

[k]Reasons for not processing samples were incomplete collection of biological sample sets from participants or poor quality of the samples

NA, not applicable; T1D, type 1 diabetes

ESM Table 2. Bacteria associated with type 1 diabetes (differentially expressed bacterial genera) identified in the assessed sample types (**A-F**). The log2FoldChange (log2FC) values show the direction of change in gravidae with type 1 diabetes/their neonates (under- or overrepresentation). All presented genera showed statistically significant variation in bacterial relative abundance (statistical significance annotations based on the Wald test with Benjamini and Hochberg correction for multiple testing).

A. Vaginal introitus (type 1 diabetes, *n*=50 vs. controls, *n*=41)

Genus	baseMean	log2FC	lfcSE	stat	*p* value	*p* adj
Gemella	7.792	21.377	1.595	13.403	5.79E-41	1.32E-38
Citrobacter	13.119	15.310	1.496	10.232	1.42E-24	1.62E-22
Terrisporobacter	2.508	14.702	1.529	9.618	6.74E-22	5.12E-20
Enhydrobacter	2.725	-5.107	0.869	-5.879	4.12E-09	2.35E-07
Klebsiella	110.863	7.791	1.361	5.726	1.03E-08	4.69E-07
Staphylococcus	11942.167	2.430	0.578	4.202	2.64E-05	0.001

B. Vaginal canal (type 1 diabetes, *n*=50 vs. controls, *n*=41)

Genus	baseMean	log2FC	lfcSE	stat	P-value	Padj
Sneathia	0.914	49.752	3.136	15.867	1.07E-56	2.44E-54
Intestinibacter	2.254	14.819	1.301	11.392	4.61E-30	5.25E-28
Atopobium	96.631	6.818	1.005	6.783	1.18E-11	8.97E-10
Megasphaera	3.664	7.796	1.382	5.640	1.70E-08	9.70E-07
Streptococcus	1247.347	-3.575	0.711	-5.031	4.89E-07	2.23E-05
Gemella	7.792	7.025	1.570	4.476	7.61E-06	2.89E-04
Prevotella_6	30.812	3.781	0.951	3.978	6.96E-05	2.27E-03

C. Cervix (type 1 diabetes, *n*=50 vs. controls, *n* =41)

Genus	baseMean	log2FC	lfcSE	stat	*p* value	*p* adj
Enhydrobacter	2.725	18.473	0.920	20.069	1.37E-89	2.03E-87
Parabacteroides	10.167	16.910	0.981	17.229	1.60E-66	1.18E-64
Collinsella	5.822	-15.187	1.051	-14.454	2.38E-47	1.17E-45
Intestinibacter	2.254	17.915	1.315	13.625	2.84E-42	1.05E-40

Business Standard

Sneathia	0.914	42.946	3.185	13.482	1.99E-41	5.88E-40
Fusicatenibacter	4.220	14.239	1.119	12.727	4.20E-37	1.04E-35
Terrisporobacter	2.508	14.197	1.529	9.287	1.58E-20	3.34E-19
Bacteroides	325.930	6.339	0.836	7.580	3.47E-14	6.42E-13
Jonquetella	1.868	15.347	2.035	7.540	4.70E-14	7.74E-13
Atopobium	96.631	6.622	1.021	6.488	8.72E-11	1.29E-09
Bifidobacterium	521.807	-5.864	0.970	-6.045	1.49E-09	2.01E-08
Anaerococcus	218.598	-3.202	0.630	-5.081	3.75E-07	4.62E-06
Gemella	7.792	7.915	1.589	4.981	6.31E-07	7.18E-06
Enterococcus	11413.427	-2.767	0.686	-4.034	5.48E-05	5.79E-04
Escherichia/Shigella	9056.504	2.793	0.731	3.820	1.33E-04	1.32E-03
Staphylococcus	11942.167	-2.088	0.580	-3.601	3.18E-04	2.93E-03
Fusobacterium	8.181	-3.910	1.202	-3.253	1.14E-03	9.94E-03

D. Rectum swabs (type 1 diabetes, *n*=44 vs. controls, *n*=40)

Genus	baseMean	log2FC	lfcSE	stat	*p* value	*p* adj
Staphylococcus	11942.167	3.133	0.569	5.505	3.69E-08	8.41E-06
Sneathia	0.914	13.857	3.134	4.421	9.81E-06	1.12E-03

E. Stool samples (type 1 diabetes, *n*=42 vs. controls, *n*=31)

Genus	baseMean	log2FC	lfcSE	stat	*p* value	*p* adj
Fusicatenibacter	4.220	26.438	1.231	21.475	2.68E-102	3.03E-100
Fusobacterium	8.181	20.087	1.353	14.849	7.10E-50	4.01E-48
Megasphaera	3.664	15.611	1.621	9.631	5.93E-22	2.23E-20
Anaeroglobus	2.731	19.463	2.233	8.716	2.88E-18	8.13E-17
Pseudomonas	5.239	7.505	0.928	8.090	5.95E-16	1.34E-14
Collinsella	5.822	-8.766	1.129	-7.765	8.16E-15	1.54E-13
Romboutsia	2.193	6.405	1.203	5.325	1.01E-07	1.63E-06

Business Standard

Peptoniphilus	132.748	3.420	0.746	4.584	4.56E-06	6.45E-05
Terrisporobacter	2.508	-7.663	1.690	-4.534	5.80E-06	7.28E-05
Sutterella	3.177	-5.191	1.197	-4.336	1.45E-05	1.64E-04
Dialister	33.951	3.499	0.957	3.654	2.58E-04	2.65E-03
Bacteroides	325.930	-3.032	0.876	-3.463	5.34E-04	5.03E-03
Actinomyces	7.449	2.885	0.849	3.398	6.79E-04	5.90E-03
Lachnoclostridium	1.616	3.845	1.188	3.236	1.21E-03	9.78E-03

F. Ear-skin swabs (type 1 diabetes, *n*=42 vs. controls, *n*=39)

Genus	baseMean	log2FC	lfcSE	stat	*p* value	*p* adj
Rothia	104.705	-7.754	0.944	-8.212	2.18E-16	4.96E-14
Micrococcus	34.245	-3.706	0.739	-5.017	5.24E-07	5.98E-05
Escherichia/Shigella	9056.504	-3.049	0.743	-4.103	4.08E-05	3.10E-03
Kocuria	7.550	-3.287	0.822	-3.999	6.35E-05	3.62E-03

Abbreviations: baseMean- the average of the normalized count values; log2FC- binary logarithm of fold change; lfcSE- standard error of log2FC; stat- statistical value of the test; *p* adj- adjusted *p* value;

Business Standard

ESM Table 3. Neonatal microbiome profiles of ear-skin swabs (type 1 diabetes, *n*=42 vs. controls, *n*=39) or stool samples (type 1 diabetes, *n*=42 vs. controls, *n*=31), evaluated by the maternal type 1 diabetes status. Presented are selected differences in Log2 median ratios when comparing type 1 diabetes versus control samples in ear-skin swabs or stool samples. Taxa displayed here have Log2 median ratios >1 and Median differences >0.001. Statistical significance annotations based on Wilcox rank-sum test followed by False Discovery Rate (FDR)-based multiple testing correction.

Sample Type	Taxon	Taxon id	Group 1	Group 2	Log_2 median ratio	Median diff	Mean diff	*p* value	Q value
Ear-skin swabs	*Acinetobacter*	od	T1D	Control	-2.472	-0.001	0.023	0.997	1.000
	Actinobacteria	as	T1D	Control	-2.533	-0.052	-0.079	0.016	0.231
	Alphaproteobacteria	be	T1D	Control	-1.900	-0.001	-0.006	0.929	0.996
	Anaerococcus	jk	T1D	Control	-1.291	-0.001	0.002	0.929	0.996
	Corynebacteriaceae	db	T1D	Control	-1.549	-0.004	-0.004	0.529	0.930
	Corynebacteriales	bo	T1D	Control	-1.608	-0.005	-0.004	0.540	0.930
	Corynebacterium	oy	T1D	Control	-1.334	-0.003	-0.004	0.597	0.930
	Gammaproteobacteria	bg	T1D	Control	-1.081	-0.018	-0.006	0.663	0.930
	Lactobacillaceae	ek	T1D	Control	1.284	0.006	-0.063	0.609	0.930
	Lactobacillales	cb	T1D	Control	-1.123	-0.048	-0.027	0.784	0.979
	Lactobacillus	je	T1D	Control	1.284	0.006	-0.063	0.609	0.930
	Micrococcaceae	dj	T1D	Control	-3.497	-0.014	-0.080	0.003	0.088
	Micrococcales	bq	T1D	Control	-3.229	-0.015	-0.075	0.006	0.125
	Micrococcus	hb	T1D	Control	-1.835	-0.001	-0.030	0.041	0.409
	Moraxellaceae	ft	T1D	Control	-2.294	-0.002	0.026	0.899	0.996
	Proteobacteria	am	T1D	Control	-1.120	-0.020	-0.012	0.580	0.930
	Pseudomonadales	ct	T1D	Control	-1.463	-0.002	0.013	0.899	0.996
	Staphylococcaceae	eg	T1D	Control	1.074	0.269	0.122	0.075	0.576
	Staphylococcus	iu	T1D	Control	1.074	0.269	0.122	0.076	0.576
	Streptococcaceae	em	T1D	Control	-1.366	-0.005	0.005	0.694	0.930
Stool samples	*Actinobacteria*	ac	T1D	Control	-2.659	-0.003	-0.014	0.034	0.601
	Bacillales	bk	T1D	Control	-1.321	-0.016	0.035	0.410	0.704
	Enterobacteriaceae	em	T1D	Control	-1.573	0.000	-0.033	0.221	0.704

Business Standard

Enterobacteriales	by	T1D	Control	-1.573	0.000	-0.033	0.221	0.704
Escherichia/Shigella	jv	T1D	Control	-1.596	0.000	-0.057	0.009	0.601
Gammaproteobacteria	aw	T1D	Control	-4.428	-0.026	-0.037	0.342	0.704
Proteobacteria	ah	T1D	Control	-4.368	-0.026	-0.037	0.354	0.704
Staphylococcaceae	dh	T1D	Control	-1.364	-0.017	0.035	0.430	0.717
Staphylococcus	gy	T1D	Control	-1.359	-0.016	0.035	0.423	0.714

Abbreviations: log2- binary logarithm; diff- difference; Q value- *p* value adjusted with FDR-based multiple testing correction

Business Standard

ESM Table 4. Influence of the maternal disease status, delivery mode, and antibiotic administration on the composition of the neonate's microflora (stool samples: type 1 diabetes, *n*=42 vs. controls, *n*=31; ear-skin swabs: type 1 diabetes, *n*=42 vs. controls, *n*=39). Beta diversity Bray Curtis adonis2 PERMANOVA analysis of VST transformed counts versus delivery mode (**A**) and antibiotics prophylaxis in the gravidae (**B**), stratified by sample type were applied. Statistical significance annotations were calculated with adonis2 PERMANOVA with permutational test (n = 9999).

A.

	Df	SumsOfSqs	MeanSqs	F.Model	R2	Pr(>F)
Disease	1	17149	17149	1.487	0.010	0.025 *
Delivery	1	23172	23172	2.009	0.013	0.001 ***
Disease:Delivery	1	11811	11811	1.024	0.007	0.226
Residuals	149	1718543	11534		0.971	
Total	152	1770674			1	

B.

	Df	SumsOfSqs	MeanSqs	F.Model	R2	Pr(>F)	
Disease	1	23963	23963	1.926	0.004	3.0E-04	***
Antibiotics	1	19261	19261	1.5482	0.003	0.001	**
Disease:Antibiotics_	1	17335	17335	1.3933	0.003	0.006	**
Residuals	506	6295371	12441		0.991		
Total	509	6355930			1		

Signif. codes: 0 ‘***’ 0.001 ‘**’ 0.01 ‘*’ 0.05 ‘.’ 0.1 ‘ ’ 1

Abbreviations: Df- degrees of freedom; Sqs- sum of squares; F.Model- statistic value of test; R2- R-squared value; Pr(>F)- the probability of observing a difference as large or larger than the one observed

Business Standard

ESM Table 5. Microbiome variation explained by all confounding variables evaluated across neonatal and maternal sample types (type 1 diabetes, *n*=50 vs. controls, *n*=41)[a].The goodness of fit statistic squared correlation coefficient (R2) used in determining the percent microbiome variation explained by a variable is calculated via multiple regression of environmental vectors and factors as dependent variables against VST transformed Euclidean sample distances ordinations axes as explanatory variables. Comparisons of significance of the correlation coefficients were tested via Monte Carlo permutational testing (n = 999).

Variable	Description	Rsquared						Pr(>r)					
		Ear	Stool	Vaginal introitus	Vaginal canal	Cervix	Rectum	Ear	Stool	Vaginal introitus	Vaginal canal	Cervix	Rectum
Type 1 Diabetes		**0.04**	0.01	0.01	0.01	0.02	**0.06**	**0.02**	0.45	0.60	0.51	0.29	**0.00**
Caesarean Delivery		0.03	0.02	0.02	0.02	0.01	0.01	0.10	0.19	0.20	0.17	0.38	0.60
Delivery Week		0.01	0.01	0.02	0.03	0.05	0.04	0.66	0.85	0.43	0.21	0.11	0.15
Birthweight	Weight (g)	0.01	0.01	0.00	0.00	0.00	0.01	0.67	0.74	0.93	0.85	0.99	0.56
	LGA	0.00	0.00	0.00	0.01	0.00	0.00	0.92	0.86	0.66	0.57	0.80	0.92
	SGA	**0.04**	0.00	0.01	0.00	0.00	**0.03**	**0.05**	0.75	0.57	0.78	0.94	**0.05**
Postnatal Feeding:	Breastfeeding	0.01	0.01	0.00	0.00	0.00	**0.07**	0.50	0.96	0.74	0.95	0.81	**0.00**
White Classification	B/C/D	**0.06**	0.01	0.00	0.01	0.01	0.03	**0.01**	0.50	0.92	0.46	0.48	0.11
	R/RF	0.00	0.00	0.01	0.00	0.00	0.03	0.87	0.27	0.52	0.81	0.96	0.07
Maternal	Pre-pregnancy BMI	0.02	0.00	0.00	0.01	**0.08**	0.01	0.43	0.97	0.89	0.57	**0.02**	0.75
	Uncontrolled 1st Trimester HbA_{1c}	0.02	0.00	0.02	0.01	0.01	0.03	0.14	0.49	0.22	0.50	0.47	0.07
	Multiparity	0.07	0.00	0.02	0.00	0.00	0.00	0.06	0.94	0.36	0.82	0.99	0.85
	Maternal Age	0.04	0.01	0.01	0.00	0.00	0.02	0.19	0.75	0.54	0.97	0.91	0.35
Probiotics	*Bifidobacterium*	**0.04**	0.00	0.00	0.00	0.00	0.00	**0.02**	0.77	0.65	0.98	0.74	0.65
	Lactobacillus	0.00	0.01	0.02	0.01	0.00	0.00	0.77	0.45	0.22	0.41	0.74	0.96

Business Standard

	Lactobacillus + *Bifidobacterium*	0.00	0.01	0.02	0.01	0.00	0.00	0.77	0.45	0.22	0.41	0.74	0.96
Comorbidities	Gestational hypertension/ preeclampsia	0.00	0.00	0.01	0.02	0.00	0.01	0.77	0.70	0.36	0.15	0.78	0.40
	Hypothyroidism	0.01	0.01	0.03	0.01	0.00	0.03	0.30	0.52	0.11	0.37	0.66	0.05
	Caesarean delivery	0.01	0.01	0.01	0.01	0.00	0.00	0.38	0.69	0.29	0.29	0.73	0.79
Antibiotics	Group B *Streptococcus*	0.01	0.13	0.01	0.02	0.02	0.00	0.26	0.06	0.58	0.24	0.14	0.99
	Premature rupture of membranes prophylaxis	0.00	0.00	0.01	0.01	0.01	0.00	0.99	0.24	0.41	0.35	0.32	0.89

Abbreviations: Pr(>F)- the probability of observing a difference as large or larger than the one observed

[a]in some comparisons the numbers were lower due to incomplete collection of biological samples sets from investigated individuals: vaginal introitus (type 1 diabetes, *n*=50 vs. controls, *n*=41), vaginal canal (type 1 diabetes, *n*=50 vs. controls, *n*=41), cervix (type 1 diabetes, *n*=50 vs. controls, *n*=41), and rectum (type 1 diabetes, *n*=44 vs. controls, *n*=40), stool (type 1 diabetes, *n*=42 vs. controls, *n*=31) and ear-skin (type 1 diabetes, *n*=42 vs. controls, *n*=39)

Business Standard

ESM Table 6. The influence of percentage of energy derived from protein (based on 24-hours dietetic recall for 7-days, received from 33 gravidae with type 1 diabetes and 14 controls) on maternal and neonatal microbiota composition.

The influence of the percentage of energy derived from protein on maternal and neonatal microbiota composition was found, regardless of the type 1 diabetes disease status (P=0.002). Also, combined, the disease and energy derived from proteins, influenced the microbiota composition (P=0.003) (beta diversity Bray Curtis adonis2 PERMANOVA analysis of VST transformed counts versus the percentage of energy derived from protein, stratified by sample type). Statistical significance annotations were calculated with adonis2 PERMANOVA with permutational test (n = 9999).

	Df	SumsOfSqs	MeanSqs	F.Model	R2	Pr(>F)	
Disease	1	23207	23207	1.941	0.007	1.0E-04	***
energy_protein_%	1	16539	16539	1.384	0.005	0.002	**
disease:energy_protein %	1	17810	17810	1.450	0.006	0.003	**
Residuals	262	3132107	11955		0.982		
Total	265	3189663			1		

Signif. codes: 0 ‘***’ 0.001 ‘**’ 0.01 ‘*’ 0.05 ‘.’ 0.1 ‘ ’ 1

Abbreviations: Df- degrees of freedom; Sqs- sum of squares; F.Model- statistic value of test; R2- R-squared value; Pr(>F)- the probability of observing a difference as large or larger than the one observed

Business Standard

ESM Table 7. Microbiota community composition in subgroups of type 1 diabetes women with HbA_{1c} ≤55 mmol/mol (*n*=39) vs. HbA_{1c} >55 mmol/mol (*n*=11), as measured in the first trimester of pregnancy. Adonis2 PERMANOVA comparisons of maternal and neonatal microbiome community beta diversity by disease state, delivery mode, and maternal first trimester HbA_{1c} values across rectum, cervix, vaginal canal, vaginal introitus, stool, and ear-skin sample types. Missing first trimester HbA_{1c} values were either imputed using a KNN based model from the remaining metadata values for type 1 diabetes participants or generated based on a norm distribution derived from average first trimester HbA_{1c} values for nondiabetic mothers (31 mmol/mol; 4.96% ± 0.53%). Statistical significance annotations were calculated with adonis2 PERMANOVA with permutational test (n = 9999).

		Weighted UniFrac				
Sample Type	Variable	SumOfSqs	R2	statistic	*p* value	
Rectum	Type 1 Diabetes	0.01	0.03	2.57	0.041	*
	Delivery Mode	0.00	0.01	0.51	0.769	
	1st Tri HbA_{1c}	0.00	0.01	0.97	0.386	
Cervix	Type 1 Diabetes	0.00	0.01	0.78	0.582	
	Delivery Mode	0.00	0.01	1.03	0.391	
	1st Tri HbA_{1c}	0.00	0.01	0.79	0.556	
Vaginal introitus	Type 1 Diabetes	0.00	0.02	1.44	0.182	
	Delivery Mode	0.00	0.02	1.67	0.112	
	1st Tri HbA_{1c}	0.00	0.02	1.75	0.095	
Vaginal canal	Type 1 Diabetes	0.00	0.01	1.14	0.323	
	Delivery Mode	0.00	0.02	1.73	0.103	
	1st Tri HbA_{1c}	0.00	0.00	0.45	0.851	
Ear-skin	Type 1 Diabetes	0.01	0.02	1.76	0.083	
	Delivery Mode	0.01	0.02	2.00	0.051	*
	1st Tri HbA_{1c}	0.01	0.04	3.20	0.008	**
Stool	Type 1 Diabetes	0.00	0.03	2.27	0.054	*

Business Standard

Delivery Mode	0.00	0.10	8.16	1.0E-04	***
1st Tri HbA_{1c}	0.00	0.01	0.63	0.634	

Abbreviations: Sqs- sum of squares; R^{2}- R-squared value

ESM Table 8. Overview of maternal microbiomes originated from vaginal introitus, vaginal canal, cervix, and rectum (considered as the microbiota 'sources' in the transfer) to microbial communities in neonates (stool samples and ear-skin swabs as 'sinks' in the transfer), evaluated by disease states (type 1 diabetes vs. controls) and delivery method (C-section vs. vaginal delivery), using SourceTracker2 and statistic values (bolded are statistically significat values based on ANOVA with a Tukey's HSD post hoc test, $p<0.05$). Mean values are in the range 0.006-0.839 (1.000 would stand for a full contribution from one source, and 0.0000 for no contribution). Statistically significant increases in maternal source rectum and decrease in cervix contributions to the ear-skin microbiome were found when comparing neonatal ear-skin microbiomes of vaginally delivered neonates of mothers with type 1 diabetes to the control women.

			Maternal source (mean ± std. error)				
Delivery	Neonatal sink	Disease state	Rectum	Cervix	Vaginal introitus	Unknown	Vaginal canal
C-section	Ear-skin	Control (*n*=19)	0.635 ± 0.31	0.022 ± 0.048	0.035 ± 0.043	0.276 ± 0.285	0.031 ± 0.051
		T1D (*n*=27)	0.691 ± 0.28	0.02 ± 0.03	0.039 ± 0.038	0.22 ± 0.25	0.031 ± 0.032
	Stool	Control (*n*=14)	0.839 ± 0.104	0.003 ± 0.003	0.067 ± 0.033	0.046 ± 0.111	0.044 ± 0.024
		T1D (*n*=26)	0.764 ± 0.252	0.003 ± 0.005	0.09 ± 0.091	0.091 ± 0.209	0.052 ± 0.069
Vaginal	Ear-skin	Control (*n*=20)	**0.595 ± 0.311**	**0.052 ± 0.092**	0.09 ± 0.096	0.18 ± 0.247	0.083 ± 0.105
		T1D (*n*=16)	**0.832 ± 0.198**	**0.007 ± 0.011**	0.051 ± 0.076	0.08 ± 0.174	0.03 ± 0.047
	Stool	Control (*n*=17)	0.653 ± 0.317	0.006 ± 0.006	0.038 ± 0.024	0.282 ± 0.331	0.021 ± 0.014
		T1D (*n*=16)	0.545 ± 0.336	0.02 ± 0.062	0.053 ± 0.053	0.346 ± 0.343	0.036 ± 0.042

Abbreviations: Std.error- standard error

Business Standard

4. ESM Figures

a.

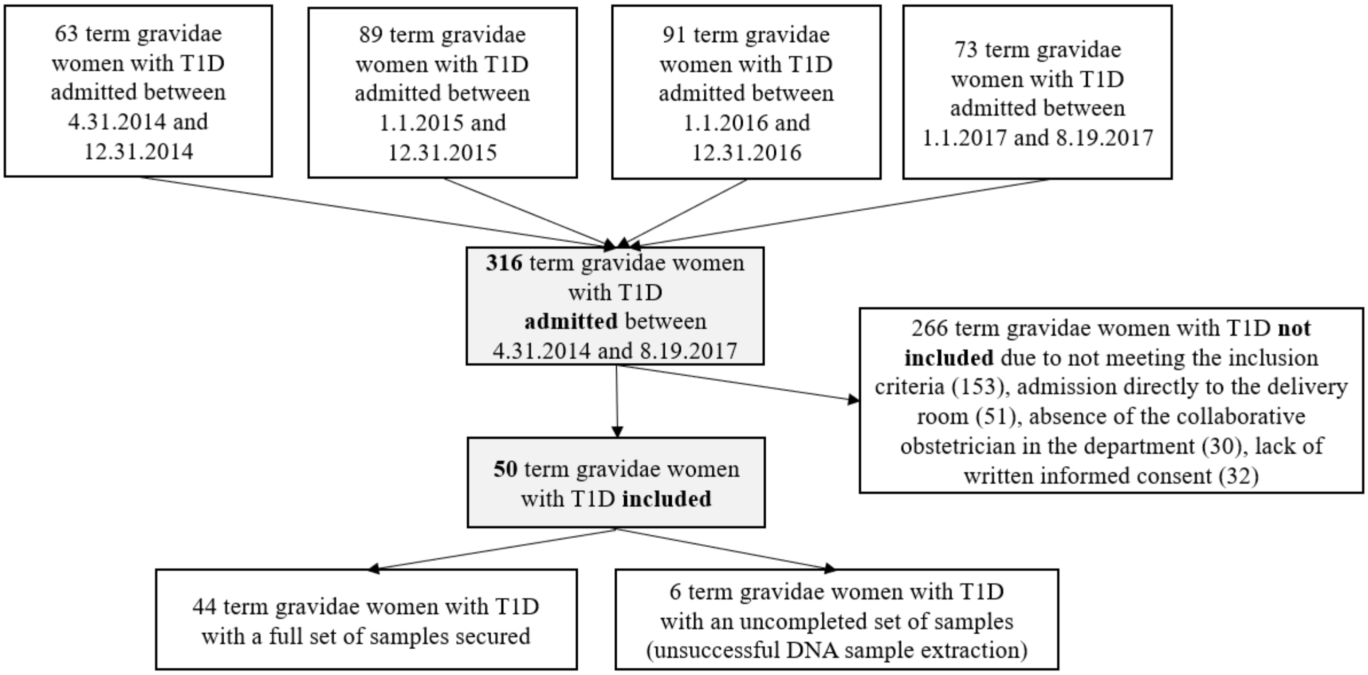

b.

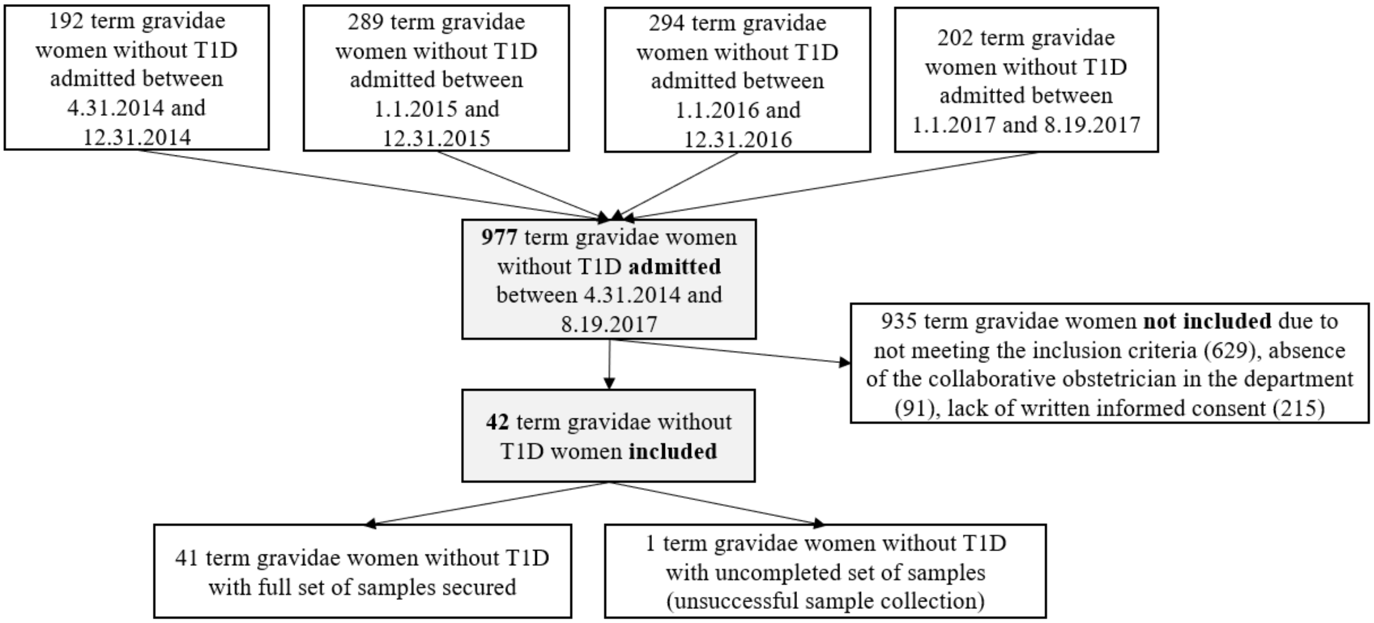

ESM Figure 1. Schemes of inclusion of participants in the study, gravidae with type 1 diabetes (**a**) and control gravidae (**b**).

a.

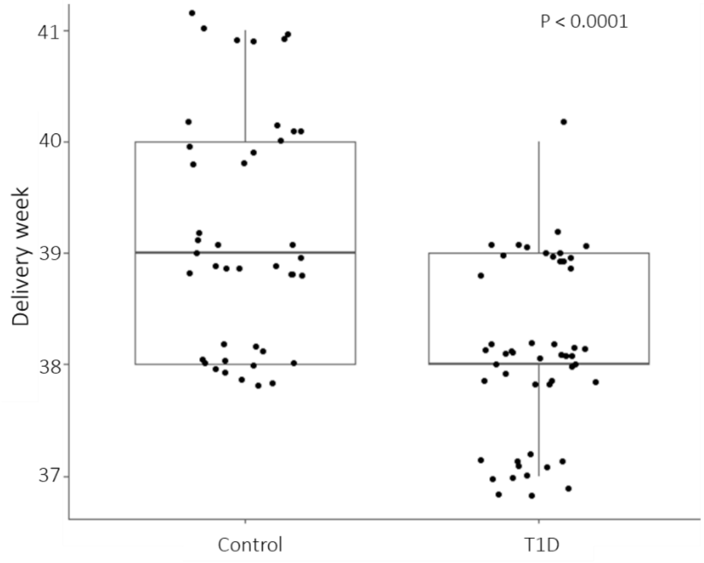

b.

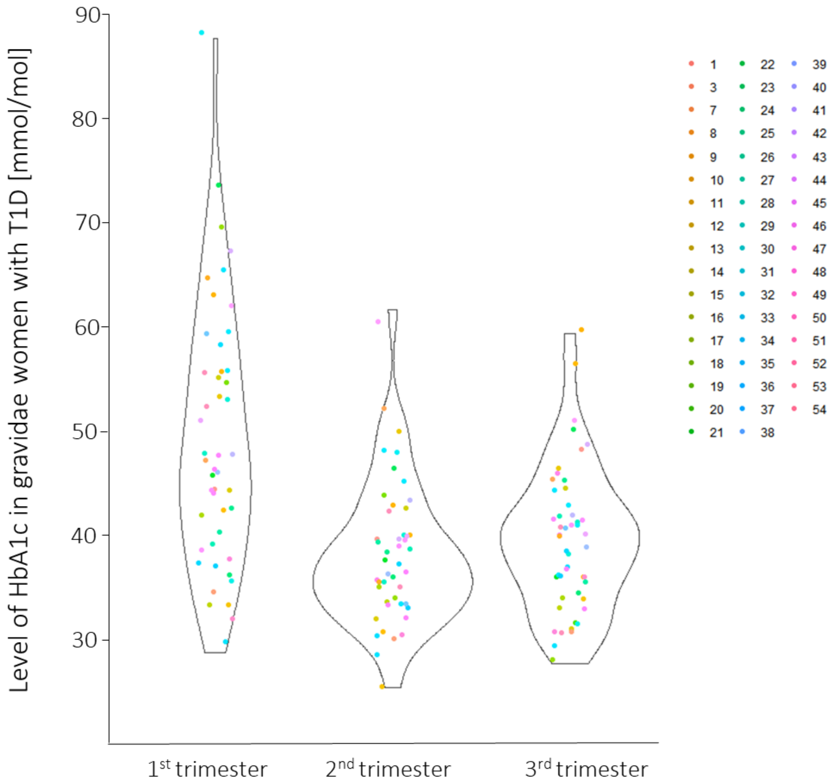

ESM Figure 2. Additional clinical findings in women with type 1 diabetes. (**a)** Disease and week of delivery. The box plot presents the differences in the gestation (weeks) between women with type 1 diabetes and control women (type 1 diabetes, n=50 vs. controls, n=42; two-tailed Mann-Whitney U Test, p<0.001). Each dot represents one woman. **(b)** The HbA_{1c} levels measured during the first, second, and third (before delivery) trimesters in gravidae with type 1 diabetes (n=50). Each dot represents one woman with type 1 diabetes. The glycemic control was found to be satisfactory in the second and third trimesters [in accordance with the recommendations of the Polish Diabetes Association, $HbA_{1c} \leq 43$ mmol/mol (≤6.1%)]. The Tukey multiple

comparisons of means show that no substantial difference in the results of diabetes control when comparing the data of the second and third trimesters of pregnancy (p= 0.682) was found.

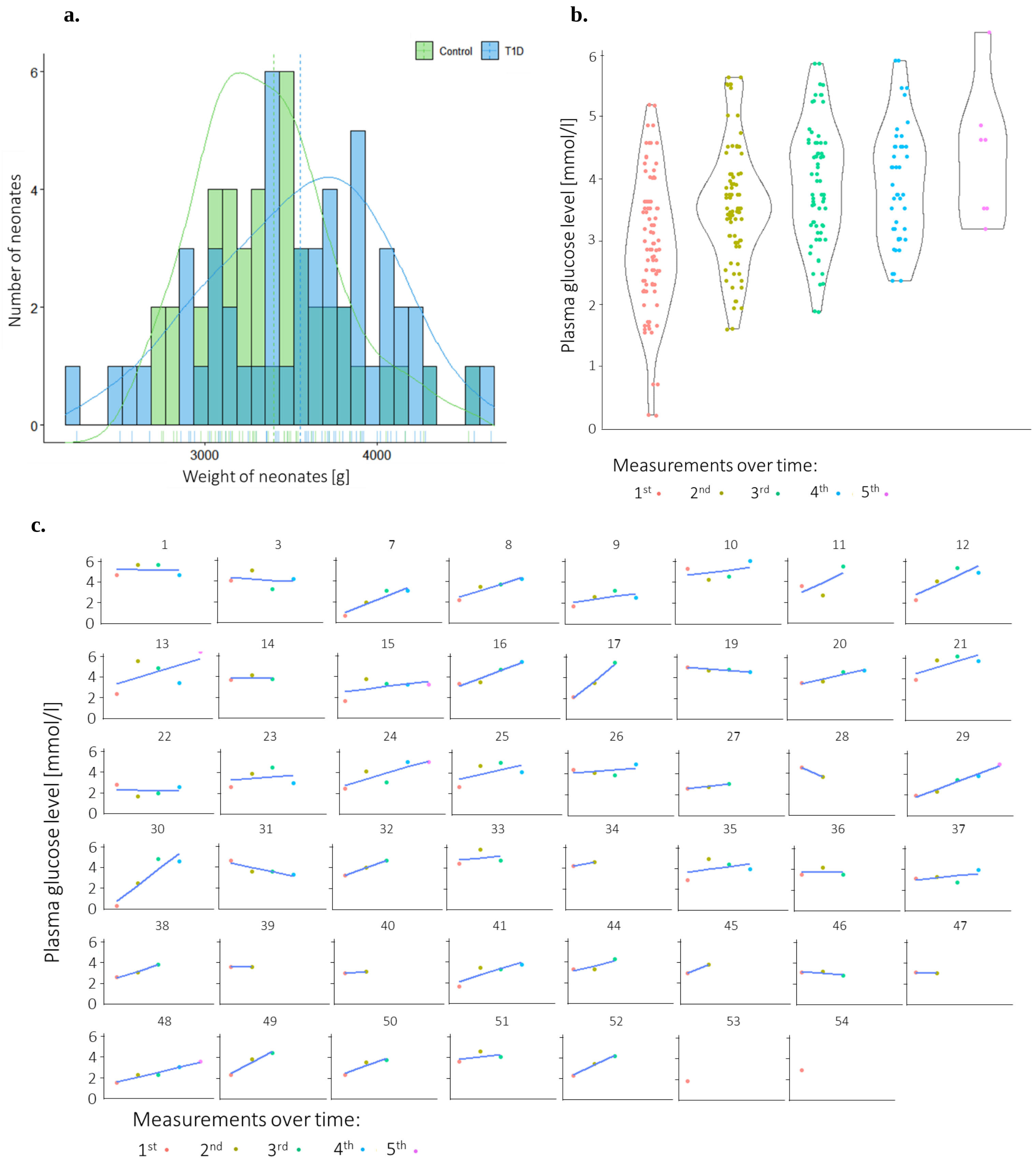

ESM Figure 3. Additional clinical findings in neonates delivered by women with type 1 diabetes (*n*=50). **(a)** Neonate weight distribution. Neonates born to mothers with type 1 diabetes had higher body weight (in grams),

Business Standard

which is indicated by the Gaussian curve shift to the right (two-tailed Mann-Whitney U Test, $p=0.095$). (**b)** The measurements of plasma glucose level [mmol/l] in two to five time points (the number of measurements depended on the condition of the neonate), performed in neonates born to women with type 1 diabetes. (**c)** Glucose level measurements over time in particular neonates of women with type 1 diabetes, presented in individual graphs.

Business Standard

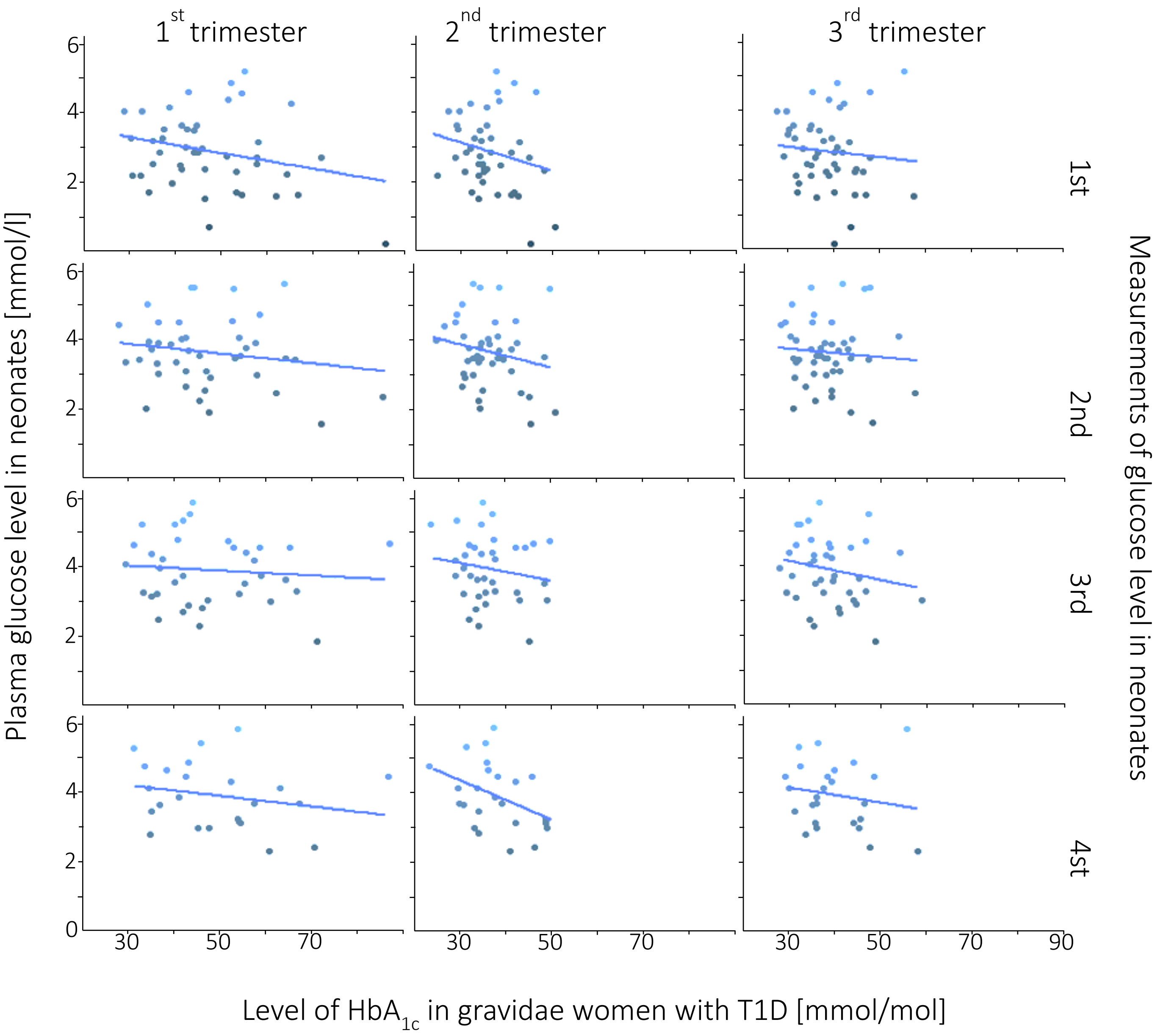

ESM Figure 4. Linear regression model for glycated hemoglobin level in gravidae with type 1 diabetes (T1D) measured before delivery, and the interaction effects of glucose measured over time in neonates born to these women (*n*=50). No correlations using Pearson's product-moment test were found regarding HbA_{1c} levels of mothers with type 1 diabetes, in the first, second, and third (delivery) trimesters, and the measurements of the glucose level in neonates ($p>0.05$).

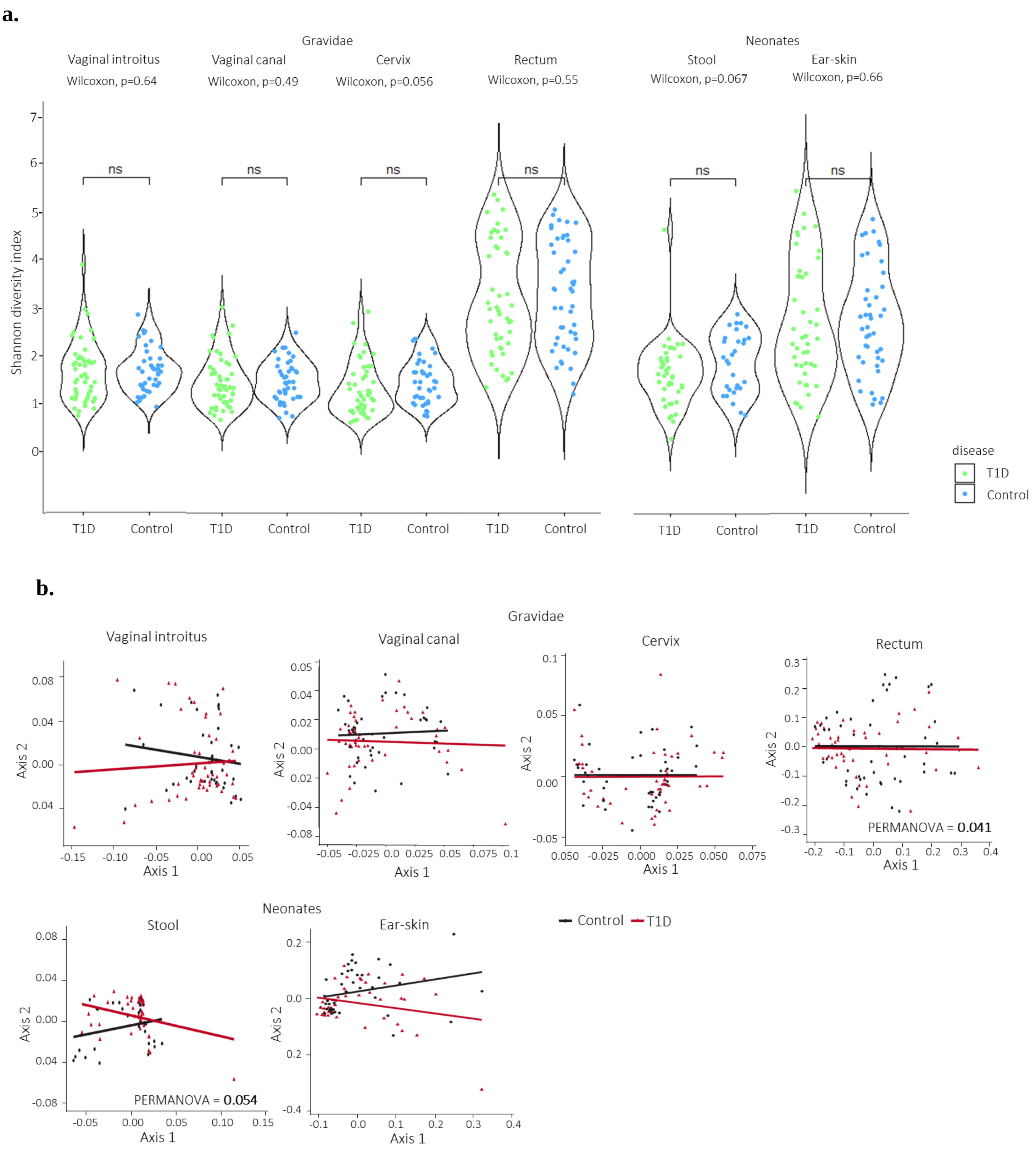

ESM Figure 5. Characterization of the maternal and neonatal microbiome using targeted 16S rRNA gene amplicon sequencing. **(a)** The alpha diversity (Shannon index) of maternal vaginal introitus (type 1 diabetes, *n*=50 vs. controls, *n*=41), vaginal canal (type 1 diabetes, *n*=50 vs. controls, *n*=41), cervix (type 1 diabetes, *n*=50

Business Standard

vs. controls, *n*=41), and rectum (type 1 diabetes, *n*=44 vs. controls, *n*=40), as well as neonatal stool (type 1 diabetes, *n*=42 vs. controls, *n*=31) and ear-skin (type 1 diabetes, *n*=42 vs. controls, *n*=39) samples. The Wilcoxon test showed no differences in bacterial abundance in terms of maternal type 1 diabetes (T1D). **(b)** The beta diversity PCoA plot comparisons of maternal and neonatal microbiome community composition by maternal type 1 diabetes in vaginal introitus, vaginal canal, cervix, and rectum as well as neonatal sample types. PCoA plots coordinates are based on Weighted UniFrac dissimilarity indices obtained from VST transformed ASV counts. Axis 1 represents the principal coordinate that explains the largest data change, and Axis 2 represents the principal coordinate that accounts for the largest proportion of the remaining data changes. Each dot represents one sample. Trendlines are derived from fitting generalized linear models for each factor and statistical significance annotations are based on adonis2 PERMANOVA with permutational test (n = 9999).

Business Standard

a.

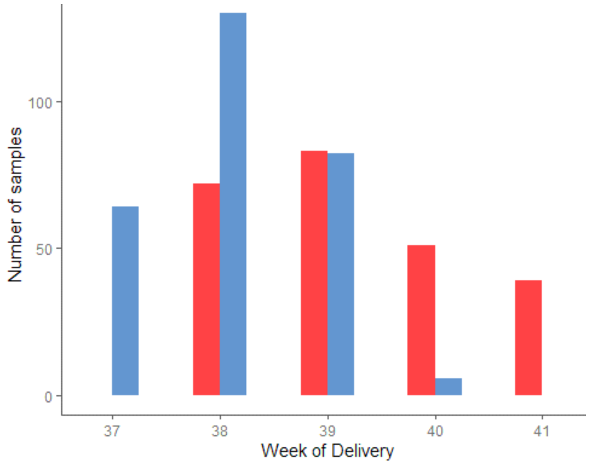

b.

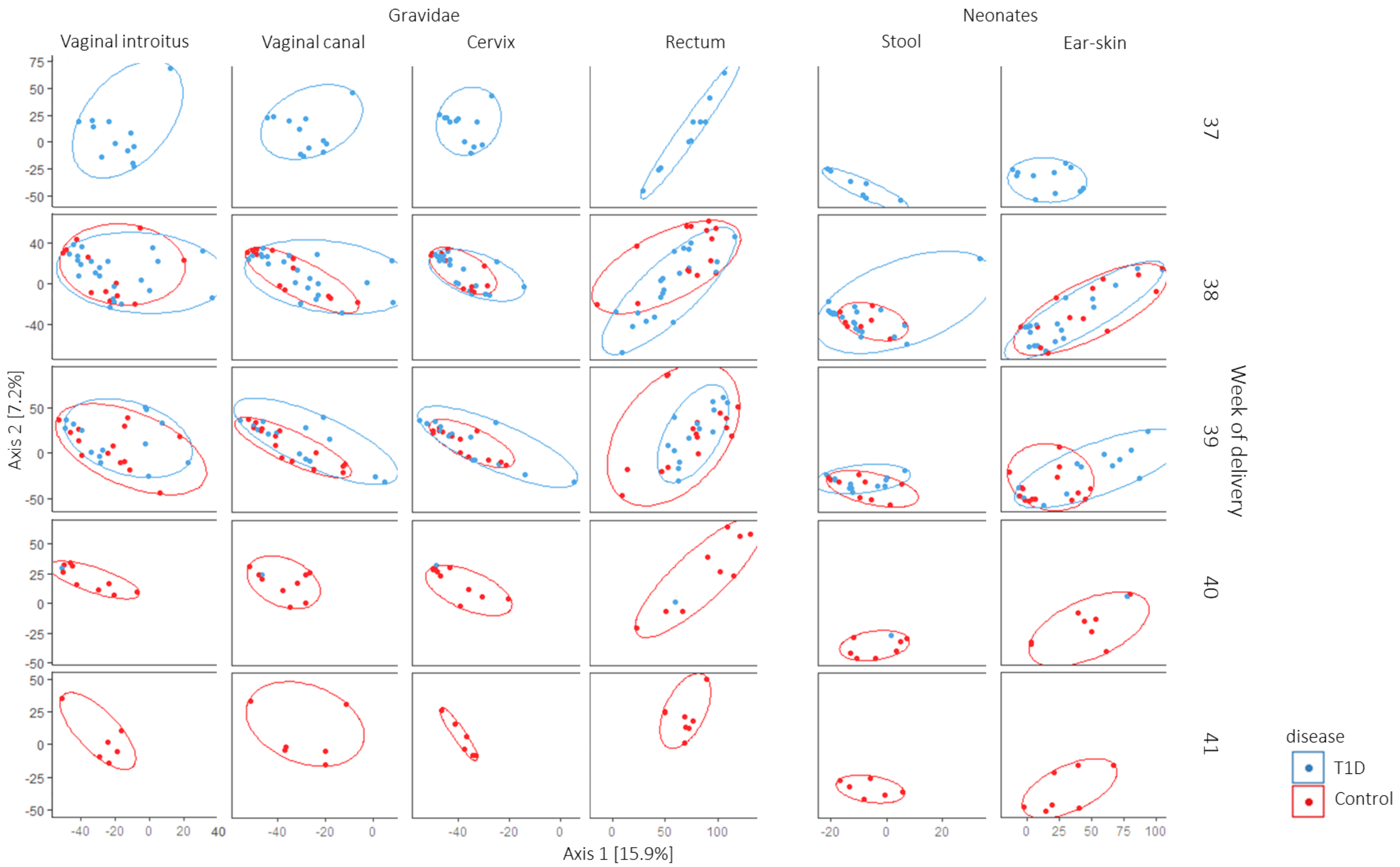

ESM Figure 6. The beta diversity PCoA plots presenting the type 1 diabetes and control samples' microbial composition across all studied sample types (vaginal introitus: type 1 diabetes, *n*=50 vs. controls, *n*=41; vaginal canal: type 1 diabetes, *n*=50 vs. controls, *n*=41; cervix: type 1 diabetes, *n*=50 vs. controls, *n*=41; rectum: type 1 diabetes, *n*=44 vs. controls, *n*=40; stool: type 1 diabetes, *n*=42 vs. controls, *n*=31; ear-skin: type 1 diabetes, *n*=42 vs. controls, *n*=39) regarding a delivery week. (**a**) The number of samples analyzed, taking into account the given week of labor. (**b**) The influence of delivery week was found to affect the microbial composition (*p*=0.002) in the assessed samples (beta diversity Bray Curtis adonis2 PERMANOVA analysis with permutational test (n = 9999), of VST transformed counts vs. week of delivery stratified by sample type). The type 1 diabetes disease status also influenced the microbial composition (*p*=0.002).

Business Standard

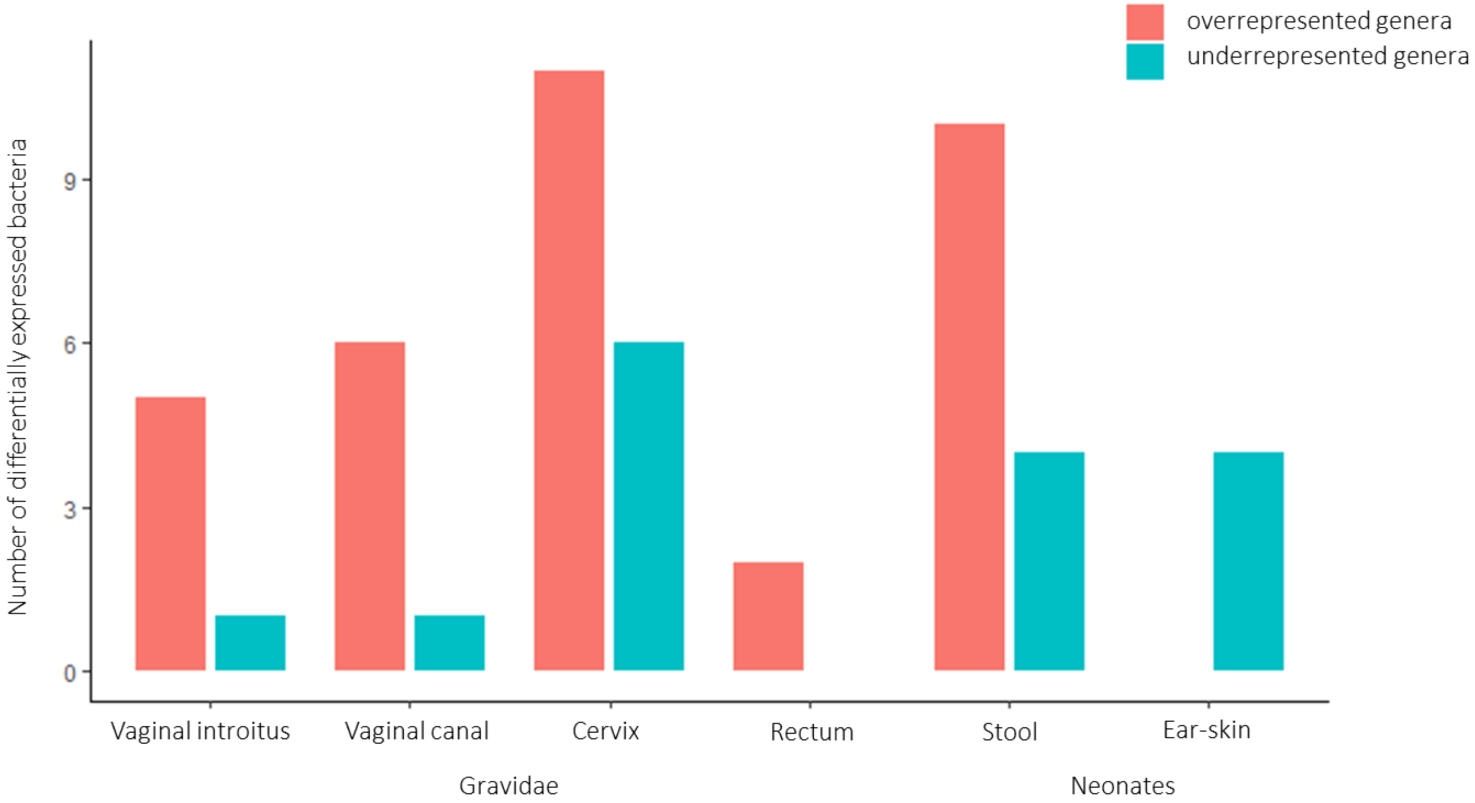

ESM Figure 7. Number of bacteria associated with type 1 diabetes (differentially expressed genera) in the maternal vaginal introitus (type 1 diabetes, *n*=50 vs. controls, *n*=41), vaginal canal (type 1 diabetes, *n*=50 vs. controls, *n*=41), cervix (type 1 diabetes, *n*=50 vs. controls, *n*=41), and rectum (type 1 diabetes, *n*=44 vs. controls, *n*=40), as well as neonatal stool (type 1 diabetes, *n*=42 vs. controls, *n*=31), and ear-skin (type 1 diabetes, *n*=42 vs. controls, *n*=39) and samples. Statistical significance annotations based on the Wald test with Benjamini and Hochberg correction for multiple testing.

Among the 17 genera, 11 were overrepresented and 6 were underrepresented in the cervix of the women with type 1 diabetes. In the vaginal introitus and vaginal canal numbers of differentially expressed bacteria associated with type 1 diabetes were similar. Two genera, *Staphylococcus* and *Sneathia* were found as overrepresented in the samples taken from rectum of the women with type 1 diabetes. Comparing the four types of material samples taken from women, *Snetahia*, *Gemella*, and *Staphylococcus as well as Intestinibacter*, *Atopobium*, *Terrisporobacter*, and *Enhydrobacter* were identified as differentially expressed genera associated with type 1 diabetes in more than one sampling site, respectively in 3 and 2 material types derived from the women with type 1 diabetes.

No overrepresented bacteria associated with type 1 diabetes in the ear-skin swabs were identified. *Rothia*, *Micrococcus*, *Escherichia/Shigella*, and *Kocuria* were underrepresented in the ear-skin swabs derived from the neonates of the women with type 1 diabetes, comparing to the ear-skin swabs derived from the neonates of the control women. *Fusicatenibacter*, *Fusobacterium*, *Megasphaera*, *Anaeroglobus*, *Pseudomonas*, *Romboutsia*, *Peptoniphilus*, *Dialister*, *Actinomyces*, and *Lachnoclostridium* were overrepresented in the stool samples derived from the neonates of the women with type 1 diabetes, while *Collinsella*, *Terrisporobacter*, *Sutterella*, and *Bacteroides* were underrepresented in these samples.

Business Standard

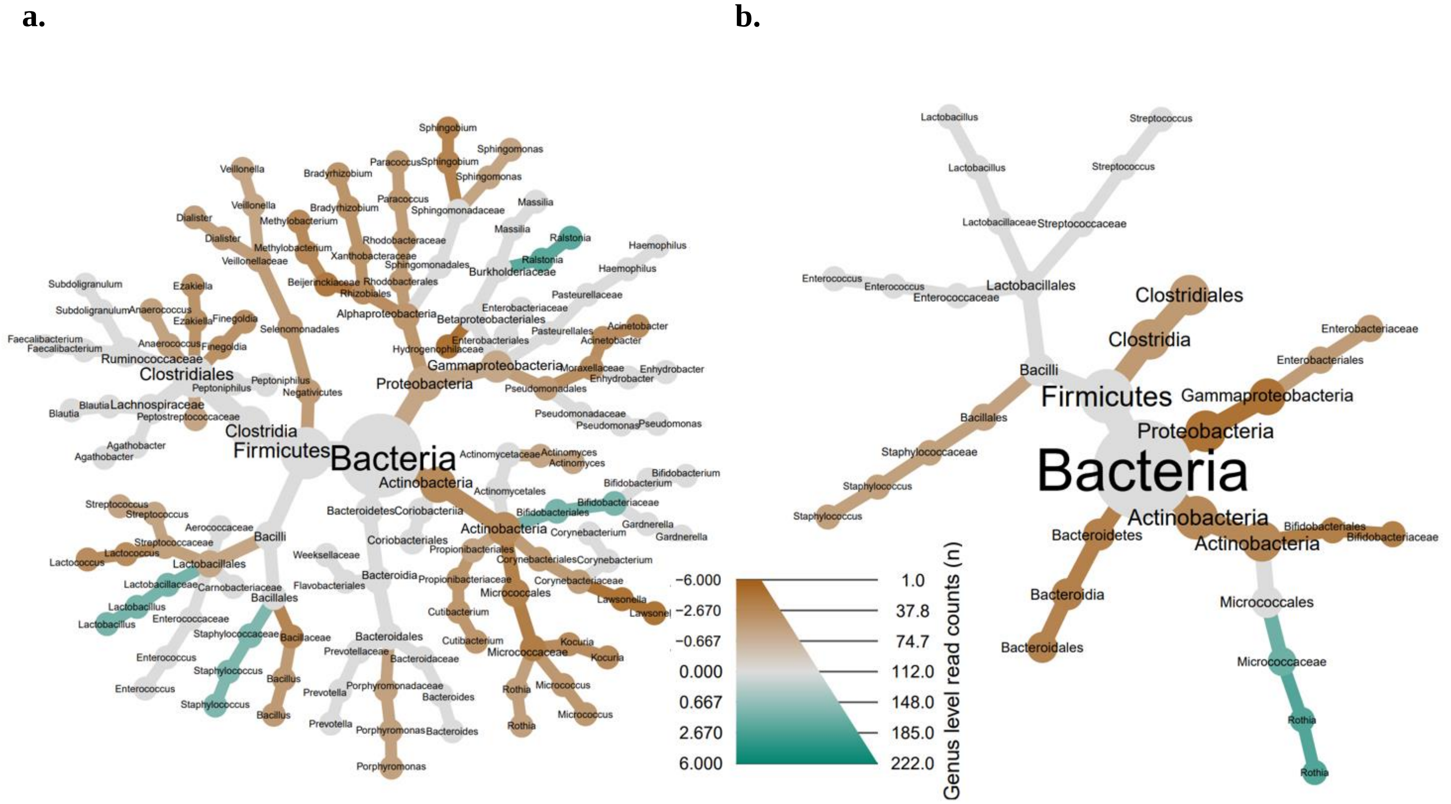

ESM Figure 8. Microbiome profiles of neonatal ear-skin swabs (type 1 diabetes, *n*=42 vs. controls, *n*=39) and neonatal stool samples (type 1 diabetes, *n*=42 vs. controls, *n*=31). Presented are heat-tree comparisons of the log2 median ratios in the neonatal microbiome profiles of ear-skin swabs (**a**) and stool samples (**b**), assessed by maternal type 1 diabetes status. Nodes are sized by the overall number of observations matching particular taxa. Nodes / edges colored in green were more prevalent among neonates of mothers with type 1 diabetes (T1D), and nodes in brown represent taxa that were more prevalent among neonates of control mothers. Statistical significance annotations based on Wilcox rank-sum test followed by False Discovery Rate (FDR)-based multiple testing correction.See ESM Table 2 for a full list of observed differences. *Micrococcaceae*, *Hydrogenophilaceae*, *Micrococcales*, *Actinobacteria*, *Lawsonella*, *Actinobacteria*, *Kocuria*, and *Micrococcus* were found to be notably decreased in ear-skin samples of neonates born to gravidae with type 1 diabetes. Also, in stool samples of neonates born to gravidae with type 1 diabetes, there were similarly decreased genera when compared to controls, but these genera were distinct and included *Escherichia/Shigella*, *Bacteroidetes*, *Bacteroidia*, *Bacteroidales*, *Actinobacteria*, *Bifidobacteriales*, *Bifidobacteriaceae*.

Business Standard

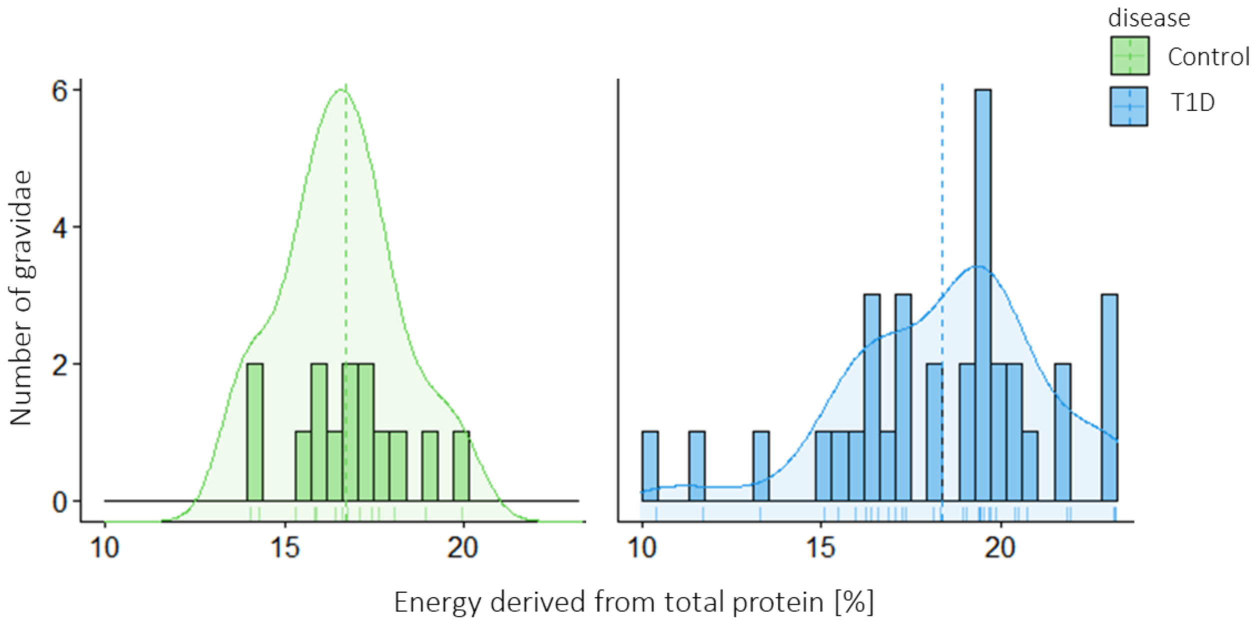

ESM Figure 9. The percentage of energy derived from protein (based on 24-hours dietetic recall for 7-days, received from 33 gravidae with type 1 diabetes and 14 controls).

The influence of the percentage of energy derived from protein on maternal and neonatal microbiota composition was found, regardless of the type 1 diabetes disease status (*p*=0.002). Also, combined, the disease and energy derived from proteins, influenced the microbiota composition (*p*=0.003) (beta diversity Bray Curtis adonis2 PERMANOVA analysis with permutational test (n = 9999), of VST transformed counts versus the percentage of energy derived from protein, stratified by sample type).

Business Standard

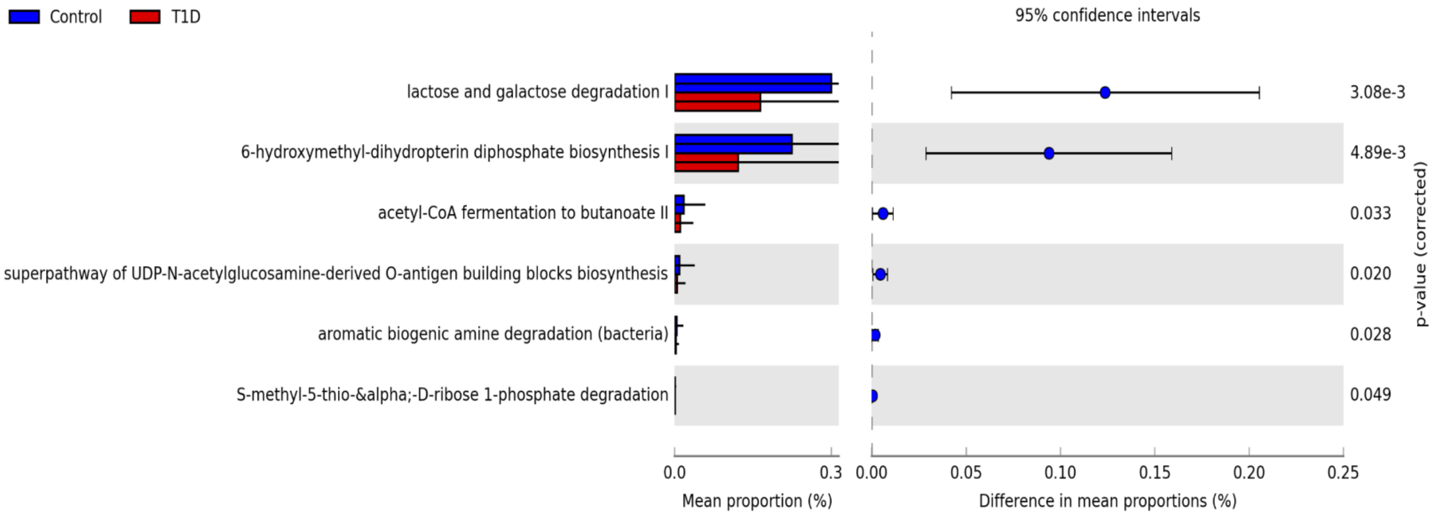

ESM Figure 10. The PICRUSt analysis of combined maternal and neonatal samples for prediction of metagenome function of their microbiomes (type 1 diabetes, *n*=50 vs. controls, *n*=41). The most underrepresented predicted pathways, 6 out of 18 recognized, in type 1 diabetes women and their neonates are presented. Statistical significance annotations based on Wilcox rank-sum test followed by False Discovery Rate (FDR)-based multiple testing correction.

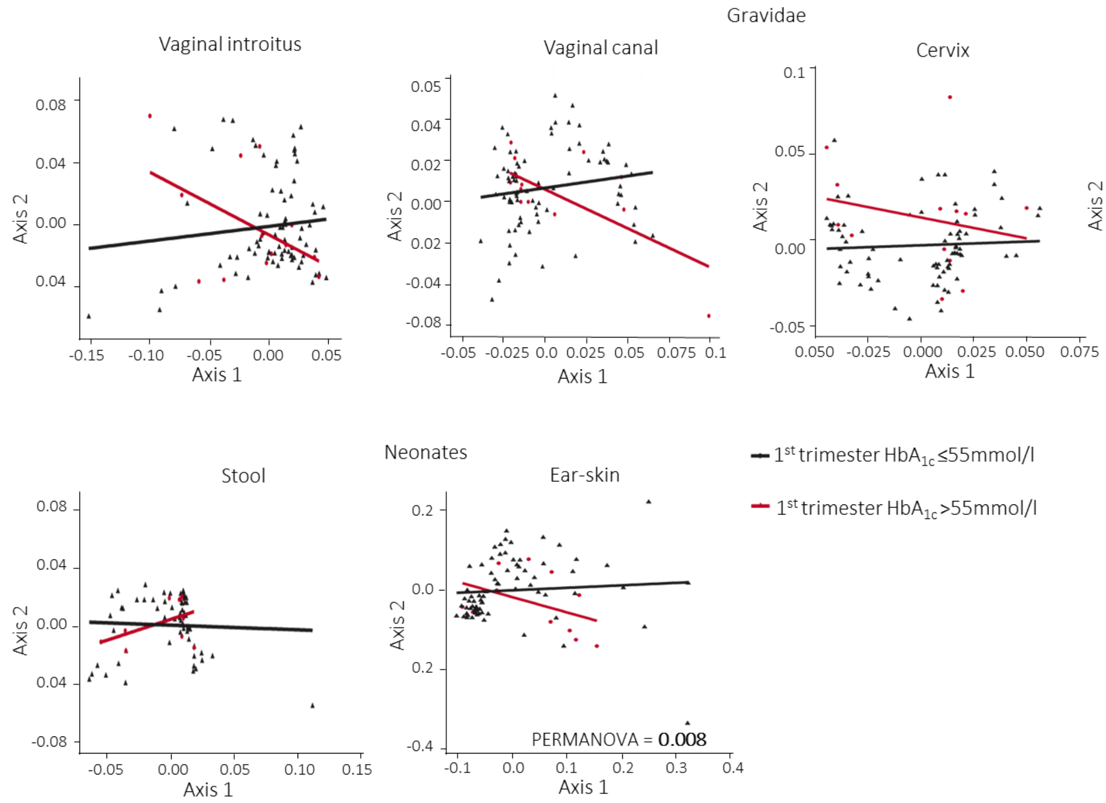

ESM Figure 11. Microbiota community composition in subgroups of type 1 diabetes women with $HbA_{1c} \leq 55$ mmol/mol (*n*=39) vs. $HbA_{1c} > 55$ mmol/mol (*n*=11), as measured in the first trimester of pregnancy, across vaginal introitus, vaginal canal, cervix and rectum as well as neonatal sample types. PCoA plots coordinates are based on Weighted UniFrac dissimilarity indices obtained from VST transformed ASV counts. Axis 1 represents the principal coordinate that explains the largest data change, and Axis 2 represents the principal coordinate that accounts for the largest proportion of the remaining data changes. Each dot represents one sample. Trendlines are derived from fitting generalized linear models for each factor and statistical significance annotations are based on adonis2 PERMANOVA with permutational test (n = 9999). See ESM Table 7 for a full list of observed differences.

Business Standard

ESM Participant Survey

Poznan University of Medical Sciences

'Microbiota in Type 1 Diabetes'

Participant Survey

Date of completing the survey ………………..................

Date of samples collection …………………....................

Study number ………………………............................

Contact phone number...

1) Name and surname ……………………..……………………......

2) Year of birth……………………………………….……..……

3) Please provide:

Height:…………………..……........……

Pre-pregnancy weight: ……….………

4) Current weight: ……………………..

**5) General diseases**:	Participant	Family	(if any, what type, since when, what medications ?)
Asthma	☐	☐	……………………………………………....
Allergy	☐	☐	……………………………………………....
Connective tissue diseases	☐	☐	……………………………………………....
Genetic diseases/syndromes	☐	☐	…………………………………………….....
Hypertension (without diabetes)	☐	☐	……………………………………………....
Cancer	☐	☐	…………………………………………….....
Hormonal disorders	☐	☐	…………………………………………….....
Diabetes	☐	☐	…………………………………………….....
Gestational diabetes (GDM)	☐	☐	…………………………………………….....

Class G1 ☐ impaired glucose tolerance with normoglycemia under dietary restrictions, treatment- diet
Class G2 ☐ fasting and postprandial hyperglycemia, requires diet and insulin treatment

Pre- gestational diabetes (PGDM)	☐	☐	…………………………………………….....
Chronic complications of diabetes:	☐	☐	…………………………………………….....
Hypertension	☐	☐	………………………………..…………...

Business Standard

Nefropathy □ □ …………………………………………...

Non-proliferative retinopathy □ □ ……………………………………….....

Proliferative retinopathy □ □ ……………………………………….....

Disorders of glucose regulation □ □ ……………………………………….....

Ishemic heart disease □ □ ……………………………………….....

Cardiomyopathy □ □ ……………………………………….....

Acidosis □ □ ……………………………………….....

Hypoglycemic episodes □ □ ……………………………………….....

Other chronic diseased □ □ ……………………………………….....

6) Do you take insulin? YES □ NO □ (since when?) ……………………………………...............

7) Do you monitor your blood sugar level? YES □ NO □

- Using a glucometer □

- Using continuous glucose monitoring system □

8) Are you on a diabetic diet? YES □ NO □ (since when, do you adhere to the diet?) ……………………………………………………………………………………………...........……....

9) Do you smoke cigarettes? (if YES, please specify how many per day) ……………………………......

10) Did you smoke cigarettes before pregnancy? ……………………………………….............……......

11) Do you take vitamins during pregnancy? ……………………...…………………............................

12) When was the last time you took antibiotics and for what reason?……………………........................

13) Have you ever undergone tretment with immunosuppressive drugs? (if YES, please specify when and for what reason) ……………………………………………………...

14) Pregnancy characteristics:

Which pregnancy?……………..………………………..……..

Which week?...

Is the course of pregnancy normal? (if NOT, please specify the type of disorder and the medications you are taking)………………………………………………………………………………………..

15) Have you ever experienced (if the cause has been diagnosed, please specify)

- Difficulty getting pregnant YES □ NO □ ……………………......
- Miscarriages YES □ NO □……………………......
- Intrauterine fetal deaths YES □ NO □ ……………………......
- Caesarean section YES □ NO □……………………......
- Preterm birth YES □ NO □……………………......
- Birth of a newborn with low birthweight YES□ NO □……………………......
- Birth of a large newborns > 4000 g YES □ NO □……………………......
- Birth of a newborn with a developmental defect YES □ NO □……………………......

- Diagnosis of diabetes in a previous pregnancy YES NO ……………………......

16) Have you ever had any illnesses or procedures in the urogenital area (if YES, please specify when and what)

……………………………………………………………………………………………….....

17) Have you ever been tested for Streptococcus agalactiae (GBS)? (if YES, please specify when and what the result was) ……………………………………………...……………………………………………………

18) Do you have a regular sexual partner?…………………………………………….......................

19) Does your partner use condoms? ……………………………………………………....................

20) Which of the following practices are / have been frequently present in your sexual life?

- Frequent changes of sexual partners
- Anal intercourse
- Oral intercourse
- Use of a vibrator

21) Do you use/have used contraceptives? (if YES, please mark which ones, and for how long)

- Hormonal pills ……………………………………………………………………….
- Hormonal patches …………………………………………………………………............
- Others (e.g., intrauterine device) …………...………………………………………………......

22) Dou you perform vaginal irrigations? NO, frequently , rarely , sporadically

23) Do you use intimate hygiene products? (if YES, please specify which ones) ……………….......

24) In the last month have you used:

- Oral or vaginal antibiotics YES
- Oral or vaginal antifungal drugs YES
- Vaginal probiotic preparations YES

25) In the last 72 hours:

- Have you performed vaginal irrigation? YES
- Have you had sexual intercourse with your partner? YES

26) Do you have information about:

- An active infection caused by Chlamydia, fungi, N. gonorrhoeae, T. vaginalis YES
- Whether you have been diagnosed with infections such as HPV, HSV-2, HIV YES

27) Do you currently have any issues related to the urogenital system (indicating an ongoing infection: discharge, itching, etc.)? YES

5. ESM References

1. Araszkiewicz A, Bandurska-Stankiewicz E, Borys S, et al (2021) 2021 Guidelines on the management of patients with diabetes. A position of Diabetes Poland. Clinical Diabetology 10(1):1–113. https://doi.org/10.5603/DK.2021.0001

2. (2008) Hyperglycemia and Adverse Pregnancy Outcomes. N Engl J Med 358(19):1991–2002. https://doi.org/10.1056/NEJMoa0707943

3. International Association of Diabetes and Pregnancy Study Groups Consensus Panel, Metzger BE, Gabbe SG, et al (2010) International association of diabetes and pregnancy study groups recommendations on the diagnosis and classification of hyperglycemia in pregnancy. Diabetes Care 33(3):676–682. https://doi.org/10.2337/dc09-1848

4. (2014) Diagnostic criteria and classification of hyperglycaemia first detected in pregnancy: A World Health Organization Guideline. Diabetes Research and Clinical Practice 103(3):341–363. https://doi.org/10.1016/j.diabres.2013.10.012

5. Wądołowska L (2005) Valldation of food frequency questionnaire (ffq). Reproducibility assessment. Bromat Chem Toksykol 38(1):27–33

6. Gutaj P, Morawska A, Kosewski G, Kamińska D, Jaśkiewicz K, Przysławski J (2020) Dietary habits of pregnant women with type 1 diabetes: do they differ from healthy controls? Pol Arch Intern Med 130(12):1107–1110. https://doi.org/10.20452/pamw.15671

7. Kunachowicz H, Przygoda B, Nadolna I, Iwanow K (2005) Tables of composition and nutritional value of food [in Polish], 1st ed. Wydawnictwo Lekarskie PZWL, Warszawa

8. Weisburg WG, Barns SM, Pelletier DA, Lane DJ (1991) 16S ribosomal DNA amplification for phylogenetic study. J Bacteriol 173(2):697–703. https://doi.org/10.1128/jb.173.2.697-703.1991

9. Callahan BJ, McMurdie PJ, Rosen MJ, Han AW, Johnson AJA, Holmes SP (2016) DADA2: High-resolution sample inference from Illumina amplicon data. Nat Methods 13(7):581–583. https://doi.org/10.1038/nmeth.3869

10. Cole JR, Wang Q, Fish JA, et al (2014) Ribosomal Database Project: data and tools for high throughput rRNA analysis. Nucl Acids Res 42(D1):D633–D642. https://doi.org/10.1093/nar/gkt1244

11. Quast C, Pruesse E, Yilmaz P, et al (2013) The SILVA ribosomal RNA gene database project: improved data processing and web-based tools. Nucleic Acids Res 41(Database issue):D590-596. https://doi.org/10.1093/nar/gks1219

12. McMurdie PJ, Holmes S (2013) phyloseq: An R Package for Reproducible Interactive Analysis and Graphics of Microbiome Census Data. PLoS ONE 8(4):e61217. https://doi.org/10.1371/journal.pone.0061217

Business Standard

13. Kolde R (2019) pheatmap: Pretty Heatmaps. R package version 1.0.12. https://cran.r-project.org/web/packages/pheatmap/index.html. Accessed 22 October 2023.

14. Oksanen J, Blanchet F G, Friendly M, et al (2020) vegan community ecology package version 2.5-7 November 2020

15. Douglas GM, Maffei VJ, Zaneveld JR, et al (2020) PICRUSt2 for prediction of metagenome functions. Nat Biotechnol 38(6):685–688. https://doi.org/10.1038/s41587-020-0548-6

16. Kassambra A (2023) ggpubr: “ggplot2” Based Publication Ready Plots. https://CRAN.R-project.org/package=ggpubr. Accessed 22 October 2023.

17. Koncevičius K (2021) matrixTests: Fast Statistical Hypothesis Tests on Rows and Columns of Matrices

18. JASP Team 2022 (2022) JASP Team (2022). JASP (Version 0.16.3)[Computer software].

19. Caspi R, Billington R, Ferrer L, et al (2016) The MetaCyc database of metabolic pathways and enzymes and the BioCyc collection of pathway/genome databases. Nucleic Acids Res 44(D1):D471–D480. https://doi.org/10.1093/nar/gkv1164

20. Huang Y-E, Wang Y, He Y, et al (2015) Homogeneity of the vaginal microbiome at the cervix, posterior fornix, and vaginal canal in pregnant Chinese women. Microb Ecol 69(2):407–414. https://doi.org/10.1007/s00248-014-0487-1

21. Ravel J, Gajer P, Abdo Z, et al (2011) Vaginal microbiome of reproductive-age women. Proc Natl Acad Sci USA 108(supplement_1):4680–4687. https://doi.org/10.1073/pnas.1002611107

22. The Human Microbiome Project Consortium (2012) Structure, function and diversity of the healthy human microbiome. Nature 486(7402):207–214. https://doi.org/10.1038/nature11234

23. Aagaard K, Riehle K, Ma J, et al (2012) A Metagenomic Approach to Characterization of the Vaginal Microbiome Signature in Pregnancy. PLoS ONE 7(6):e36466. https://doi.org/10.1371/journal.pone.0036466

24. MacIntyre DA, Chandiramani M, Lee YS, et al (2015) The vaginal microbiome during pregnancy and the postpartum period in a European population. Sci Rep 5:8988. https://doi.org/10.1038/srep08988

25. Pace RM, Chu DM, Prince AL, Ma J, Seferovic MD, Aagaard KM (2021) Complex species and strain ecology of the vaginal microbiome from pregnancy to postpartum and association with preterm birth. Med 2(9):1027–1049. https://doi.org/10.1016/j.medj.2021.06.001

26. Mejía-León ME, López-Domínguez L, Aguayo-Patrón SV, Caire-Juvera G, Calderón de la Barca AM (2018) Dietary Changes and Gut Dysbiosis in Children With Type 1 Diabetes.

Journal of the American College of Nutrition 37(6):501–507. https://doi.org/10.1080/07315724.2018.1444519

27. Fan Y, Pedersen O (2021) Gut microbiota in human metabolic health and disease. Nat Rev Microbiol 19(1):55–71. https://doi.org/10.1038/s41579-020-0433-9

28. Lavelle A, Sokol H (2020) Gut microbiota-derived metabolites as key actors in inflammatory bowel disease. Nat Rev Gastroenterol Hepatol 17(4):223–237. https://doi.org/10.1038/s41575-019-0258-z

29. Vatanen T, Kostic AD, d'Hennezel E, et al (2016) Variation in Microbiome LPS Immunogenicity Contributes to Autoimmunity in Humans. Cell 165(4):842–853. https://doi.org/10.1016/j.cell.2016.04.007

30. Aagaard KM (2020) Mode of delivery and pondering potential sources of the neonatal microbiome. EBioMedicine 51:102554. https://doi.org/10.1016/j.ebiom.2019.11.015

31. Helman S, James-Todd TM, Wang Z, et al (2020) Time trends in pregnancy-related outcomes among women with type 1 diabetes mellitus, 2004-2017. J Perinatol 40(8):1145–1153. https://doi.org/10.1038/s41372-020-0698-x

32. Roth-Schulze AJ, Penno MAS, Ngui KM, et al (2021) Type 1 diabetes in pregnancy is associated with distinct changes in the composition and function of the gut microbiome. Microbiome 9(1):167. https://doi.org/10.1186/s40168-021-01104-y

33. Bertani B, Ruiz N (2018) Function and Biogenesis of Lipopolysaccharides. EcoSal Plus 8(1):ecosalplus.ESP-0001-2018. https://doi.org/10.1128/ecosalplus.ESP-0001-2018

34. Xu F, Tavintharan S, Sum CF, Woon K, Lim SC, Ong CN (2013) Metabolic signature shift in type 2 diabetes mellitus revealed by mass spectrometry-based metabolomics. J Clin Endocrinol Metab 98(6):E1060-1065. https://doi.org/10.1210/jc.2012-4132

35. Li Y, Chen JP, Duan L, Li S (2018) Effect of vitamin K2 on type 2 diabetes mellitus: A review. Diabetes Res Clin Pract 136:39–51. https://doi.org/10.1016/j.diabres.2017.11.020

36. Ceccarani C, Foschi C, Parolin C, et al (2019) Diversity of vaginal microbiome and metabolome during genital infections. Sci Rep 9(1):14095. https://doi.org/10.1038/s41598-019-50410-x

37. Wang J, Xu J, Han Q, et al (2020) Changes in the vaginal microbiota associated with primary ovarian failure. BMC Microbiol 20(1):230. https://doi.org/10.1186/s12866-020-01918-0

38. Stewart CJ, Ajami NJ, O'Brien JL, et al (2018) Temporal development of the gut microbiome in early childhood from the TEDDY study. Nature 562(7728):583–588. https://doi.org/10.1038/s41586-018-0617-x

39. Sakwinska O, Foata F, Berger B, et al (2017) Does the maternal vaginal microbiota play a role in seeding the microbiota of neonatal gut and nose? Benef Microbes 8(5):763–778. https://doi.org/10.3920/BM2017.0064

40. Wender-Ożegowska E, Bomba-Opoń D, Brązert J, et al (2018) Standards of Polish Society of Gynecologists and Obstetricians in management of women with diabetes. Ginekol Pol 89(6):341–350. https://doi.org/10.5603/GP.a2018.0059
